# Differential epigenetic reprogramming in response to specific endocrine therapies promotes cholesterol biosynthesis and cellular invasion

**DOI:** 10.1038/ncomms10044

**Published:** 2015-11-27

**Authors:** Van T. M. Nguyen, Iros Barozzi, Monica Faronato, Ylenia Lombardo, Jennifer H. Steel, Naina Patel, Philippa Darbre, Leandro Castellano, Balázs Győrffy, Laura Woodley, Alba Meira, Darren K. Patten, Valentina Vircillo, Manikandan Periyasamy, Simak Ali, Gianmaria Frige, Saverio Minucci, R. Charles Coombes, Luca Magnani

**Affiliations:** 1 Department of Surgery and Cancer, Imperial College London, London W12 0NN, UK; 2 IFOM-IEO Campus, European Institute of Oncology, Milan 20139, Italy; 3 School of Biological Science, University of Reading, Reading RG6 6LA, UK; 4 MTA TTK Lendület Cancer Biomarker Research Group, 2nd Department of Pediatrics, Semmelweis University, Budapest H-1117, Hungary; 5 MTA-SE Pediatrics and Nephrology Research Group, 2nd Department of Pediatrics, Semmelweis University, Budapest H-1117, Hungary; 6 ECMC, Charing Cross Hospital, London W120nn, UK; 7 Department of Pharmacy, Health and Nutritional Sciences, University of Calabria, Arcavacata di Rende (CS) 87036, Italy

## Abstract

Endocrine therapies target the activation of the oestrogen receptor alpha (ERα) via distinct mechanisms, but it is not clear whether breast cancer cells can adapt to treatment using drug-specific mechanisms. Here we demonstrate that resistance emerges via drug-specific epigenetic reprogramming. Resistant cells display a spectrum of phenotypical changes with invasive phenotypes evolving in lines resistant to the aromatase inhibitor (AI). Orthogonal genomics analysis of reprogrammed regulatory regions identifies individual drug-induced epigenetic states involving large topologically associating domains (TADs) and the activation of super-enhancers. AI-resistant cells activate endogenous cholesterol biosynthesis (CB) through stable epigenetic activation *in vitro* and *in vivo*. Mechanistically, CB sparks the constitutive activation of oestrogen receptors alpha (ERα) in AI-resistant cells, partly via the biosynthesis of 27-hydroxycholesterol. By targeting CB using statins, ERα binding is reduced and cell invasion is prevented. Epigenomic-led stratification can predict resistance to AI in a subset of ERα-positive patients.

## Abstract (web summary)

How breast cancer cells adapt to individual therapies targeting the oestrogen receptor alpha is poorly understood. Here the authors show resistance emerging through differential epigenetic reprogramming that activates the cholesterol biosynthesis pathway.

## Graphical abstract

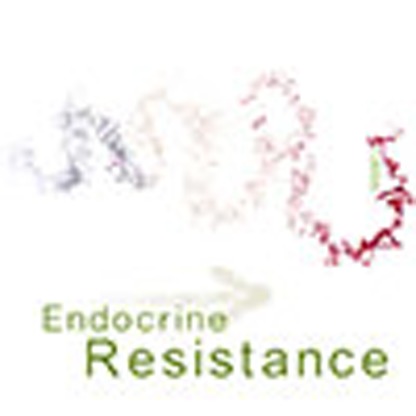

Over 70% of all breast cancers are characterized by the expression of the nuclear receptor oestrogen receptor alpha (ERα)^1^. Patients with ERα-positive disease are routinely treated with adjuvant endocrine therapies (ETs) after surgery. ETs include a series of compounds designed to interfere with ERα activation, including selective oestrogen receptor modulators (for example, tamoxifen), aromatase inhibitors (AIs, for example, Letrozole and Anastrozole) and selective oestrogen receptor downregulators (Faslodex/Fulvestrant)^2^. Tamoxifen competitively bind to ERα in the place of estrogens such as estradiol (E2). AIs are designed to deplete the estrogens in circulation, while Fulvestrant irreversibly bind to ERα leading to ERα degradation. Over 40% of ERα breast cancers eventually relapse from ETs becoming progressively refractory to further treatments^1^. It remains unclear whether resistance to ETs involves drug-specific mechanisms. Since ETs are characterized by different mechanisms^2^,^3^, it is conceivable that chronic exposure to therapy may impart specific selective pressure and elicit different response mechanisms to induce stable phenotypic changes.

During development and differentiation, cell identity is established by the epigenetic activation of cell-type-specific distal regulatory elements mediated by combinatorial patterns of histone modifications^4^,^5^. These regions are often embedded in chromatin-accessible sites and contain DNA sequence motifs for cell-type-specific transcription factors (TFs)^6^. The epigenome retains some degree of plasticity, as cell identity can be epigenetically reprogrammed by exposure to external stimuli such as the ectopic expression of TFs^6^. Cancer cells can also remodel histone modifications and chromatin accessibility in response to chronic exposure to therapeutic agents^7^,^8^,^9^. For example, adaptation to oestrogen deprivation in ERα-positive breast cancer cells invokes extensive epigenetic reprogramming especially in distal regulatory regions such as enhancers^7^. Regulatory elements directly associated with active transcription, and more complex regulatory regions (that is, super-enhancers (SEs)) can be mapped by measuring the histone modification histone 3-lysine 27 (H3K27ac)^10^,^11^,^12^,^13^,^14^. In this study, we have investigated the nature of drug adaptation to a diverse range of ETs in isogenic ERα-positive breast cancer cells using integrative and orthogonal epigenomic approaches.

Our data demonstrate that breast cancer cells evolve individual epigenomic and transcriptomic profiles in response to treatment. Both *in vitro* and *in vivo*, AI-resistant cells acquire the most aggressive phenotype and develop invasive characteristics in two-dimensional and three-dimensional (3D). Integrative analyses of AI-resistant cells identify stable upregulation of the entire cholesterol biosynthesis (CB) pathways including genes involved in 27-hydroxyl-cholesterol (27HC) biosynthesis. 27HC stimulation is sufficient to promote oestrogen-independent ERα binding to thousands of putative regulatory regions. In agreement with this finding, chromatin immunoprecipitation sequencing (ChIP-seq) analysis of the chromatin of AI-resistant cells confirmed extensive ERα binding despite oestrogen-deprived conditions. Furthermore, CB blockers (statins) can reduce ERα binding to DNA and abrogate cell invasion. CB upregulation also occurs *in vivo* during breast cancer progression. Finally, we demonstrate that a CB-based signature might be used to improve the stratification of ERα breast cancer patients before adjuvant treatment.

## Results

### Adaptation to AI treatment leads to *de novo* invasiveness

**Figure 1:**
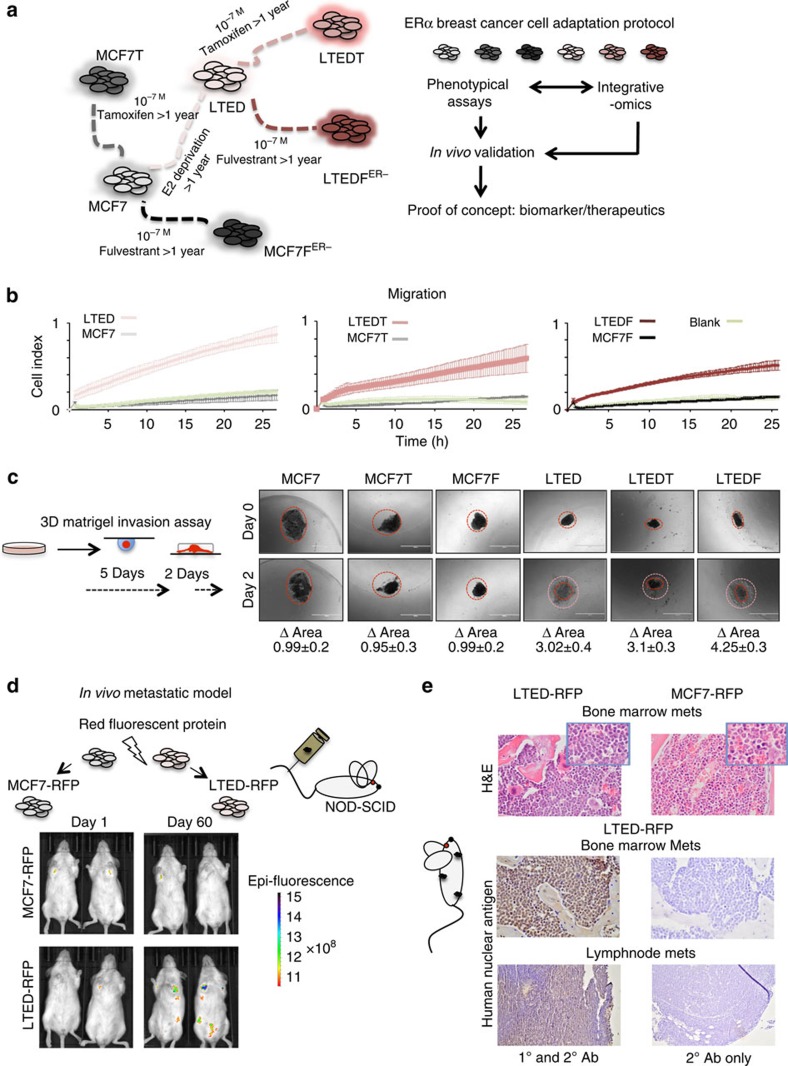
Adaptation to AI treatment is associated with cell invasion. (**a**) *In vitro* adaptation experimental protocol: ERα-positive breast cancer cells (MCF7) were chronically exposed to endocrine therapies to generate ETR lines^15^. Each cell type was screened for the development of invasive potential. ETR cells were also profiled using integrative epigenomics and the results were cross-validated using clinical samples. (**b**) Real-time monitoring of cell migration demonstrates increased motility of LTEDs cells compared with other resistant lines. (**c**) 3D organoids assay was used to demonstrate invasive potential under physical constraints (Matrigel). Δ Area is calculated using ImageJ (area day 2/area day 0, *n*=5). The horizontal white bar indicates a distance of 1,000 μm. (**d**) LTED cells can form metastases *in vivo* at various sites. Cells were stably transfected with the mCherry construct, injected in the tail of the mice and followed using *in vivo* imaging. (**e**) Snapshots of histological analysis of tissues from mice injected with LTED or MCF7 cells. The first two panels represent haematoxylin and eosin (H&E) staining on bone marrow (insets taken at a higher magnification). The bottom four panels represent immunohistochemistry on tissue from mouse bone marrow or lymph nodes showing invasion by cells immunoreactive for human nuclear antigen (left panels) or negative controls with no primary antibody (right panels).

ETs are designed to block oestrogen-driven proliferation by interfering with one specific TF (for example, ERα). However, we hypothesized that the development of resistance may follow distinct routes and generate alternative phenotypes through the different molecular mechanisms specific to each agent^2^. To test this hypothesis, we used a series of isogenic cell lines resistant to single agents or a combination of agents (endocrine therapy (ET)-resistant ETR cells, Fig. 1a)^15^. Our aim was to understand the connection between the acquisition of drug-resistance and breast cancer progression, particularly metastatic development. We then carried out a real-time, impedance-based assay to monitor the migratory and invasion behaviour of ETR cells. These assays demonstrated that long term estrogen deprived (LTED) cells (mimicking AI resistance^16^) specifically develop migration and invasion properties, while MCF7- and TAM/Fulvestrant-resistant cells (MCF7T and MCF7F) do not (Fig. 1b; Supplementary Fig. 1A). It is worth noting that sequential resistance did not increase these traits (LTEDT and LTEDF versus LTED). To corroborate these findings, we developed a 3D invasion assay (organoids assay) in which the cells were allowed to form 3D bodies and then embedded in Matrigel. In agreement with the 2D assay, AI-resistant cells (LTEDs) spread remarkably through the Matrigel environment, while the MCF7s do not (Fig. 1c; Supplementary Movie 1A–D). Finally, we developed a metastatic mouse model to validate our findings *in vivo* by engineering two red-fluorescent protein reporter (mCherry) lines (MCF7-FRP and LTED-RFP). After injection into the tail vein of NOD-SCID mice (Fig. 1c), only LTED cells colonize several sites including the lymph nodes and bones (Fig. 1d). Histology analyses of putative metastatic tissues from the lymph nodes and bone marrow confirms that the invading cells are of human origin and contains breast cancer protein markers^17^ (HNA and PBX1 (ref. 18), Fig. 1d; Supplementary Fig. 1B). Overall, these data demonstrate that AI-resistant cells acquire traits common to aggressive breast cancers. Tamoxifen- or Fulvestrant-resistant cells, although becoming resistant to the cytostatic effect of the agent, do not evolve into highly invasive cells thus supporting the notion of agent-specific reprogramming.

### ETR cells follow distinct reprogramming routes

To decipher the molecular changes induced by drug-specific resistance, we developed an unbiased integrative approach combining RNA sequencing (RNA-seq) and H3K27ac ChIP-seq in ETR cells. All ETR cells express significantly lower messenger RNA (mRNA) levels for several oestrogen receptor target genes, indicating that ET treatments still exert negative pressure on ERα signalling. As expected, MCF7F and LTEDF cells acquire an ERα-negative status, while the rest of the panel remained ERα positive (Supplementary Fig. 2A). Interestingly, other chromatin components of ERα signalling^19^ (for example, ERα pioneer factors such as FoxA1, PBX1 and GATA3 (refs 20, 21)) remain expressed at similar levels in all cell lines (Supplementary Fig. 2B,C). Cell lines with sequential resistance are characterized by a greater number of differentially regulated genes (Supplementary Fig. 3A). Correspondingly, these cells display increased numbers of potential regulatory regions as demonstrated by the significant increase in H3K27ac-positive loci (Supplementary Fig. 3B). This increase could not simply be attributed to general changes in H3K27ac distribution (Supplementary Fig. 3C).

**Figure 2:**
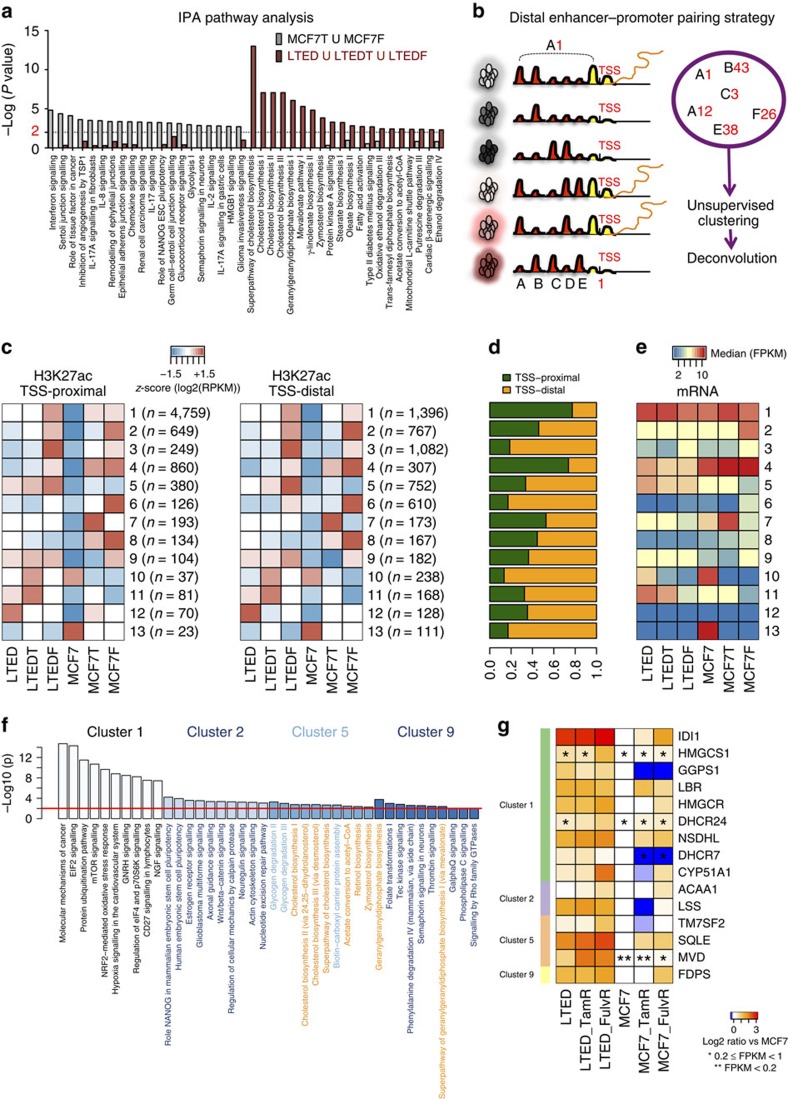
Drug adaptation and epigenetic reprogramming. (**a**) IPA pathway analysis of differentially upregulated genes (for details of the analysis, see Supplementary Fig. 4A). The top 20 IPA pathways for invasive and non-invasive cells are plotted with their respective *P* values. Metabolic pathways are in bold. The dotted line is set at a pVal=0.01. (**b**) Diagrammatic image of the bioinformatics strategy adopted for the epigenetic analysis in the six cell lines. For more details, please see the Methods (**c**) Heatmaps showing the mean H3K27ac level in each cluster for each of the six cell lines. Regions were split into TSS-proximal and distal and shown as separate heatmaps. Total number of regions in brackets. (**d**) The proportion of TSS-proximal and distal regions in each cluster is shown. (**e**) Heatmap showing the median of the linear FPKM for each cluster along the cell lines. (**f**) IPA canonical pathways significantly enriched considering the genes putatively regulated by the TSS-proximal elements in enhancer clusters. The 10 most enriched terms were extracted and their log10 (*P* value) (Fisher's exact test) are shown as bar plots. The red line is set at a pVal=0.01. (**g**) FPKM values of genes belonging to the ‘Superpathway of Cholesterol Biosynthesis' (IPA canonical pathways) identified using H3K27ac patterns alone but also showing a significant deregulation in RNA-seq compared with MCF7. Transcripts showing low expression levels are indicated with asterisks.

We then carried out a gene ontology analysis on our RNA-seq data to identify potential pathways responsible for the invasive phenotype characterizing LTED cells. To increase the power of these analyses, we clustered together all the genes differentially regulated in invasive (LTED–LTEDT–LTEDF) and non-invasive (MCF7T–MCF7F) cells (Supplementary Fig. 4A). Strikingly, we found that LTED cells activated metabolic pathways and lipid metabolism (for example, super-pathway of CB, Fig. 2a; Supplementary Fig. 4B; Supplementary Data 1). More importantly, these pathways were not active in non-invasive MCF7T and MCF7F cells (grey versus red bars), suggesting differential transcriptional reprogramming (Fig. 2a). These changes are not transiently imposed by the culture condition, but rather represent stably engrained transcriptional. In point of fact, we could not re-establish the original transcriptional profile (for example, MCF7) by culturing resistant cells in MCF7 culture conditions and the absence of endocrine treatment (Supplementary Fig. 4C).

We hypothesized that the reprogrammed transcriptomes evolved by invasive LTEDs cells might play a central role in driving aggressive breast cancer *in vivo*. To test this, we devised five individual cell signatures containing the top 10% of upregulated genes specific to each individual cell line (Supplementary Data 2) and split patients into high and low expressors (Supplementary Fig. 5A). Analysis of the METABRIC^22^ data set (restricting it to the ERα-positive patients) demonstrates that only LTED-based signatures significantly predict poor survival (Supplementary Fig. 5B,C). Collectively, these data demonstrate that AI-resistant cell lines evolve transcriptional programmes typical of patients developing more aggressive tumours.

### Drug resistance invokes activation of regulatory elements

We reasoned that chronic drug exposure probably acts as a constant selective pressure within our cell line system and allow for selective activations of regulatory regions. Indeed, we demonstrated that these changes become heritable and eventually independent from media conditions. We then investigated the epigenetic circuitry that drives the cell-type-specific transcription in our ETR models. By comparing our epigenomic maps with publically available H3K27ac profiles, we confirmed the common ancestry of ETR cells (data from ENCODE^5^), suggesting that adaptation to therapy does not entirely upset the epigenetic landscape (Supplementary Fig. 6A). Next, we devised a strategy to pinpoint the distal regulatory enhancers actively involved in regulating cell-type-specific gene expression (Fig. 2b). We first identified all the loci that showed changes in H3K27ac by comparing ETR cells with MCF7s using MACS^23^. We then pooled and clustered all the regions showing a significant increase or decrease in at least one cell line according to the co-variation of their patterns across the six cell lines (Fig. 2b and extended Methods). Using this strategy, we extrapolate genome-wide groups of potentially interacting TSS and enhancer pairs sharing the same pattern of activity across the lines (13 different clusters). Each cluster is then de-convoluted to TSS-proximal and TSS-distal (canonical enhancers) elements (Fig. 2c,d). We then match the promoter-proximal H3K27-acetylated regions to RefSeq genes and annotate the genes according to our RNA-seq analysis. These analyses demonstrate that H3K27ac at distal enhancers is highly consistent with H3K27ac at promoter and mRNA transcription (Fig. 2e). Of note, our analysis provides a solid alternative for studying distal regulatory regions in the absence of detailed interaction maps.

We then used Ingenuity Pathway Analysis to identify cell-type-specific pathways based on canonical enhancers (Supplementary Data 3). Cluster 1 contains mainly promoter-proximal elements (Fig. 2d) and shows an increase in H3K27ac signals in all resistant cells (Fig. 2c). In agreement, pathways significantly enriched in this cluster have been previously associated with ET resistance (mTOR, EIF2, NGF, EGF and HER2 signalling^22^,^24^) (Fig. 2f). Clusters 2 and 3 are directly associated to the loss of ERα, thus demonstrating the central role of this TF in breast cancer cells. Despite over half of the regions in these groups mapping to distal enhancers, coding regions unambiguously assigned to TSS-proximal elements are enriched for pluripotency genes (Fig. 2f), a feature typical of basal, stem-like ERα-negative breast cancers^25^. Focusing on LTED-specific regulatory elements, cluster 5 and cluster 9 are almost exclusively enriched for genes belonging to the super-pathway of CB confirming our transcriptional analysis (Fig. 2f). Overall, these unbiased epigenomics analyses strongly support our initial findings, and substantiate the hypothesis that AI-resistant cells epigenetically activate distinct pathways compared with other resistant cells.

### Drug-resistance-specific activation of SEs

**Figure 3:**
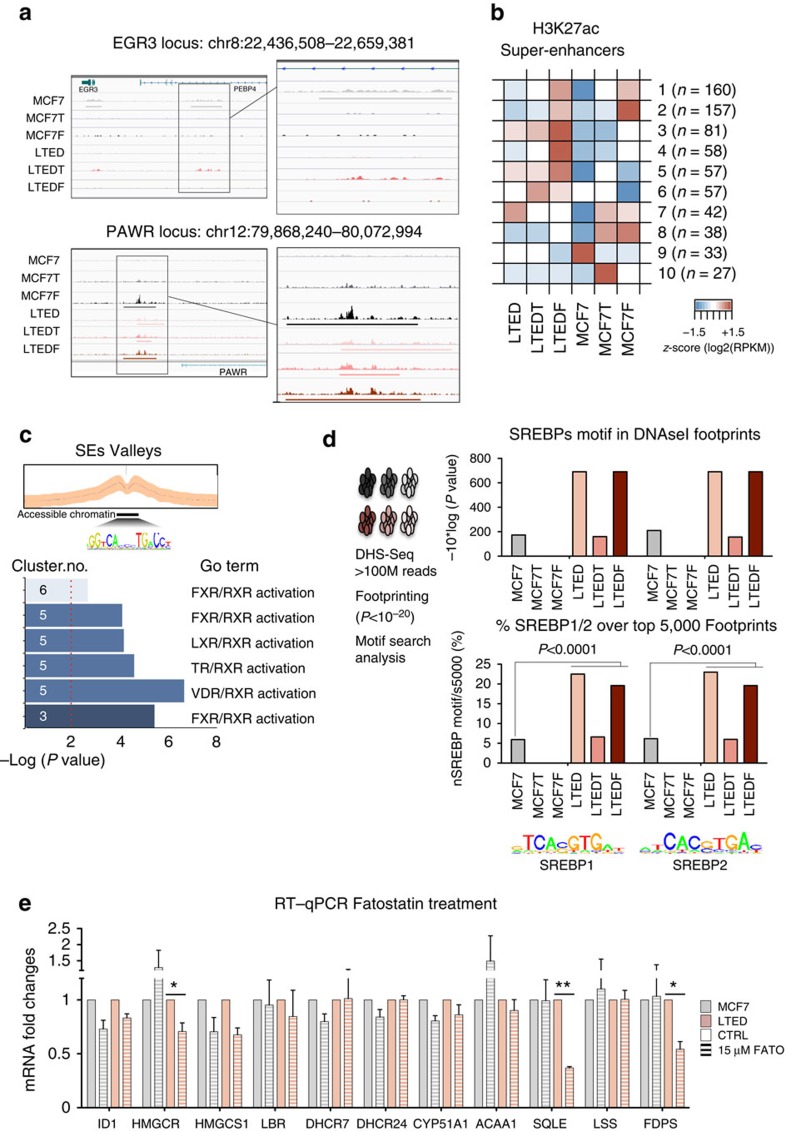
Activation of super-enhancers during epigenetic reprogramming. (**a**) Snapshot representing SE peaks and associated raw data for two genomic locus underlying cell-type-specific phenotypes (EGR3 and PAWR). (**b**) Heatmaps showing the mean H3K27ac level in each cluster of SE regions for each one of the six cell lines. Total number of regions in brackets. (**c**) DNA motif analysis (HOMER) was carried out in the potential regulatory regions between H3K27ac-marked nucleosomes for specific clusters of SEs. Gene Ontology analysis based on potential interacting TFs is shown in the bottom panel. The vertical dashed red line indicates *P*=0.01. (**d**) Pipeline for the analysis of DHS-set Footprinting data sets. SREBP1 and SREBP2 footprints are uniquely enriched within the accessible chromatin landscape of LTEDs cells. The left panel indicates *P* values from Cistrome analysis (Sitepro). The right panel displays the number of motifs within the top 5,000 footprints (expressed as a percentage). Statistical analyses were carried out using a Pearson's *χ*^2^-test. (**e**) Treatment with a SREBP1 inhibitor (Fatostatin) induces downregulation of key cholesterol biosynthesis transcripts specifically in LTED cells. Error bars represent s.e.m. calculated on three independent experiments. Asterisks indicate significant statistical differences comparing treated with mock-treated cells (*T*-test, *P*<0.05).

Our integrative epigenomic analyses suggest that endogenous activation of CB is one of the key pathways epigenetically activated in AI-resistant cells. Epigenetic activation of the CB pathway appears to occur through a multi-step process. For example, many regulatory elements associated with genes belonging to CB are included in cluster 1 (common to all resistant cells, Supplementary Data 3), indicating that these genes are active also in Tamoxifen- or Fulvestrant-resistant cells (Fig. 2g). Nevertheless, the rate-limiting enzymes of this pathway and their associated regulatory regions (for example, HMGCR and SQLE) are ultimately activated/upregulated only in oestrogen-deprived LTED cells (Fig. 2g). We therefore decided to investigate whether epigenomic activation of CB involves more complex epigenetic features such as SEs. Recent publications^11^,^26^,^27^ have identified dense clusters of distal regulatory regions that are strongly associated with transcription and particularly susceptible to perturbation, termed SEs (Fig. 3a). Adapting a previously published approach^27^, we identified a set of 710 SEs with different types of distribution across ETR cells (Supplementary Fig. 6B). Initial clustering analysis using SEs reveals that LTEDs cells share extensive epigenetic features. Instead, MCF7F cells maintain fewer connections with parental MCF7 further demonstrating the marked effect of ERα loss (Supplementary Fig. 6C). As expected, SEs activation strongly reflects cell unique traits, for example, the SEs associated with oestrogen/oestrogen receptor target gene^17^ EGR3 was identified only in MCF7 cells (Fig. 3a). Next, we clustered SEs to obtain 10 distinct groups using a similar approach-based on co-variance (Figs 2b and 3b and extended materials). IPA analysis of LTED-specific SE clusters (clusters 3 and 5) identified once more metabolic and signalling pathways. It must be pointed out that the acetylation of SEs was strongly associated with the expression of nearby genes, in agreement with previous findings^28^ (Supplementary Fig. 6D; Supplementary Data 4). Finally, we identified the TF motifs buried within peak-valley-peak structures to gain further insights into the regulatory circuitry underlying these SEs. Running IPA analysis to identify pathways coordinated by TFs that could potentially bind these SE valleys, we found that LTED-specific SE clusters 3, 5 and 6 were enriched for several metabolic nuclear receptor-driven pathways (Fig. 3c, the TFs identified include FOXO1, FOXA1, FOXA2, NR5A2 for cluster 3 and RXRG, VDR, RXRB, RXRA for cluster 5). Finally, we investigated the accessible chromatin landscape to gain more detailed insights into global changes in TF occupancy using high-depth DHS-seq^29^. Footprinting analysis to scan for putative binding sites using DHS-seq identifies a massive increase in SREBP1 and SREBP2, the two master regulators of cholesterol homeostasis in mammals^30^, in LTED cells (Fig. 3d). Significantly, SREBP1/e are also the top candidates transcriptional regulators in LTED for our RNA-seq-based analysis (see Supplementary Data 1, upstream regulators). SREBP1 activates cholesterol genes by binding their promoter after nuclear translocation in response to intracellular cues^31^,^32^. SREBP1 nuclear translocation can be inhibited using a novel series of compounds called fatostatin^33^. To test whether SREBP1 translocation was effectively one of the key events in CB activation, we treated MCF7 and LTED cells with fatostatin. Fatostatin treatment induced a significant reduction for several key genes in the CB pathway including the two rate-limiting enzymes HMGCR and SQLE specifically in LTED cells (Fig. 3e).

In summary, SEs and chromatin accessibility analyses further demonstrate a role for cholesterol-driven signalling centred on the potential crosstalk between nuclear receptors and cholesterol-specific TFs. These data suggest that epigenetic reprogramming might induce new transcription factor dependencies.

### Epigenetic reprogramming involves larger chromatin structures

**Figure 4:**
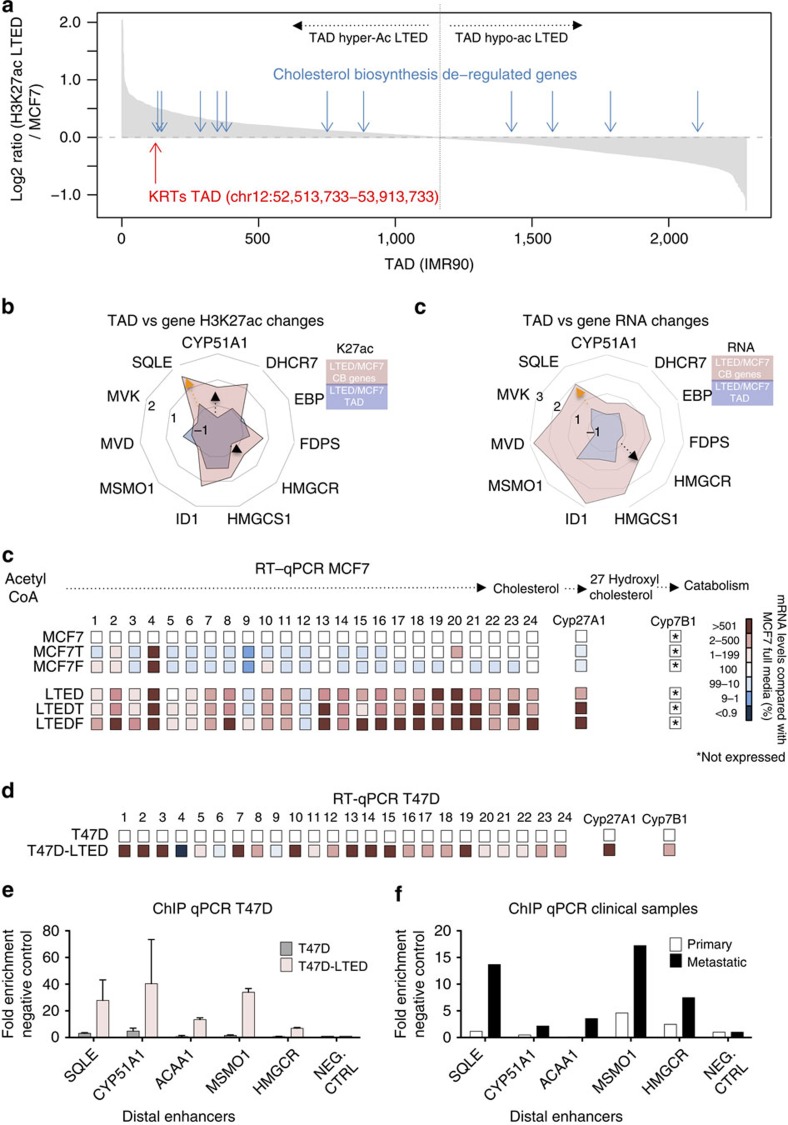
Epigenetic activation of cholesterol biosynthesis. (**a**) In all, 2,283 topologically associating domains (TADs) (identified in IMR90 cells^33^ and showing acetylation in at least one out of the six cell lines) were sorted according to the ratio of their H3K27ac levels in LTED versus MCF7. The red arrow highlights the TADs encompassing the KRT genes (chr12:52,513,733−53,913,733) while the light-blue arrows highlights the TADs encompassing the deregulated genes in the cholesterol biosynthesis pathway. (**b**) Radar plots of global (TAD) versus local (CB genes) changes in H3K27ac and RNA transcription. Data are represented as ratios (Log2 RPKM LTED/RPKM MCF7). (**c**) RT–qPCR analysis of all the enzymes involved in cholesterol and 27HC biosynthesis (identifiers in Supplementary Fig. 8A). RNA fold differences were calculated comparing MCF7 with individual resistant cells averaging at least three independent experiments. All comparisons with different colours were statistically significant (*T*-test). (**d**) RT–qPCR analysis of all the enzymes involved in cholesterol and 27HC biosynthesis (identifiers in Supplementary Fig. 8A). RNA fold differences were calculated comparing T47D with individual resistant cells averaging at least three independent experiments. (**e**) ChIP–qPCR for H3K27ac demonstrates strong enrichment at distal regulatory regions controlling CB genes in AI-resistant T47D cells. Error bars represent s.e.m. calculated on three independent experiments. (**f**) ChIP–qPCR for H3K27ac demonstrates strong enrichment at distal regulatory regions controlling CB genes in metastatic clinical samples.

Recent evidence suggests that SEs are regulated in the context of TADs^28^, with relatively insulated effects within CTCF-defined regions. TADs are physically defined genomic regions that shows cohesive gene regulation during development^33^. Several studies indicate that TAD boundaries are generally resistant to differentiation^34^. We observed that in some instances, clusters of genes physically located in the same genomic region (for example, keratin locus, chr12q13.13) are differentially regulated as a single transcriptional unit (Supplementary Data 4). More specifically, the KRT locus (KRT7, KRT80, KRT81, KRT83 and KRT86) is upregulated in all resistant cells and its putative SE belongs to cluster 1 (Fig. 3b). This raises the possibility of coarse-grained epigenetic reprogramming occurring at the level of entire genomic compartments. To test this hypothesis, we sorted 2,283 TADs (previously identified in IMR90 cells^33^) according to the ratio of their H3K27ac levels in LTED versus MCF7 (Fig. 4a). The TAD encompassing the KRT genes (chr12:52,513,733−53,913,733) ranked in the top 5%, showing a 1.42-fold increase in H3K27ac in LTED cells (Fig. 4a), confirming that epigenetic reprogramming might target sets of genes simply based on their physical proximity.

We then investigated whether analogously to KRTs, CB genes are regulated in response to coarse-grain activation of their respective SE-TAD structures. Genes in the CB pathways were found scattered over different genomic compartments with no coherent changes in H3K27ac, suggesting a TAD-independent, punctuated regulation (Fig. 4a). Thus, while some CB genes might be passively regulated in response to widespread epigenetic changes, others may be controlled by more precise epigenetic changes, often counteracting the local TAD changes. For example, at the SQLE locus the local increase in acetylation (LTED versus MCF7) substantially overshadows the modest increase in TAD acetylation (Fig. 4b, yellow arrow). Strikingly, genes such as HMGCR and CYP51A1 gain H3K27ac signals in their putative regulatory regions despite locally losing acetylation at the TAD level (Fig. 4b, black arrows). We obtained similar results when comparing global changes in RNA transcription within the TAD to the local changes for single CB genes. These data strongly suggest that CB genes activated in non-invasive cells (Fig. 2g) might be passengers of TAD-wide epigenetic activation, while rate-limiting CB genes are specifically targeted in AI-resistant cells only.

To further investigate the role of CB in AI-resistant cells, we transcriptionally profiled the entire cholesterol superpathway using reverse transcription (RT)–quantitative (q)PCR. The CB pathway is made up of 24 genes coding for enzymes necessary to build cholesterol molecules from acetyl-derived carbons (Fig. 4c; Supplementary Fig. 7A). Strikingly, 22/24 genes in the CB superpathway are upregulated in LTED cells, while 2/24 and 3/24 are upregulated in MCF7T and MCF7F cells, respectively (Fig. 4c). To rule out cell-type-specific bias in the activation of CB, we then analysed a second ERα-positive cell lines (in T47D breast cancer cells^35^). In agreement with MCF7 data, we observe a marked upregulation of CB transcripts in T47D-LTED cells as compared with naive T47D (21/24, Fig. 4d). On the other hand, non-tumorigenic MCF10A have much lower mRNA levels for all CB genes compared with non-invasive T47D or MCF7 cells (average 14±11%).

To confirm the epigenetic nature of CB activation in T47D-LTED cells, we assessed the H3K27ac status of several regulatory elements comparing resistant and naive cells. In agreement with MCF7 ETR cells, T47D-LTED exhibit a strong increase in K27acetylaiton at enhancers associated with CB genes (Fig. 4e). Finally, we investigated the epigenetic activation of distal enhancers associated with CB directly in clinical specimens. In agreement with our *in vitro* data, ChIP–qPCR analysis demonstrate a significant increase in H3K27ac signal in a biopsy derived from a metastatic deposit (AI resistant) compared wih a sample derived from a primary, drug-sensitive ERα breast cancer patient (Fig. 4g).

Altogether, these data demonstrate that the selective pressure operated by chronic drug exposure is counteracted using very distinctive epigenetic mechanisms. More specifically, these data strongly support the centrality of CB epigenetic activation in AI-resistant cells.

### CB promotes ERα activation

ERα and other nuclear receptors can be activated by a vast array of ligands including sexual hormones (estrogens) and other cholesterol-derived compounds. Recent evidence has shown that cholesterol derivatives such as 25 and 27 hydroxycholesterol can also promote the transcription of ERα target genes in breast cancer cells^36^,^37^. Interestingly, 27HC can also increase metastatic invasion in mice xenografted with MCF7 cells^38^. We then hypothesized that one of the possible consequence of epigenetic activation of CB in AI-resistant cells might be the activation of autocrine signalling via *de novo* synthesis of 27HC^39^. To test this, we initially investigated the expression levels of CYP27A1 and CYP7B1, the two enzymes involved in the synthesis and catabolism of 27HC from cholesterol precursors^39^. Analogously to CB genes, CYP27A1 is significantly upregulated in LTEDs compared with MCF7 and other resistant cells (Fig. 4c). Similarly, T47D-LTED exhibited an increase in CYP27A1 compared with parental T47D cells (Fig. 4d). On the other hand, we could not identify CYP7B1 transcripts in our cells (Fig. 4c,d). CYP27A1 is highly expressed in macrophages but also in ERα-positive breast cancer cells *in vivo*^38^. For example, we could identify strong expression of CYP27A1 in two newly established ERα metastatic cell line models derived from pleural effusion of AI-resistant patients (Supplementary Fig. 7B). These data suggest that LTED cells can convert the cholesterol obtained via augmented CB into 27HC in an autocrine pattern^39^.

**Figure 5:**
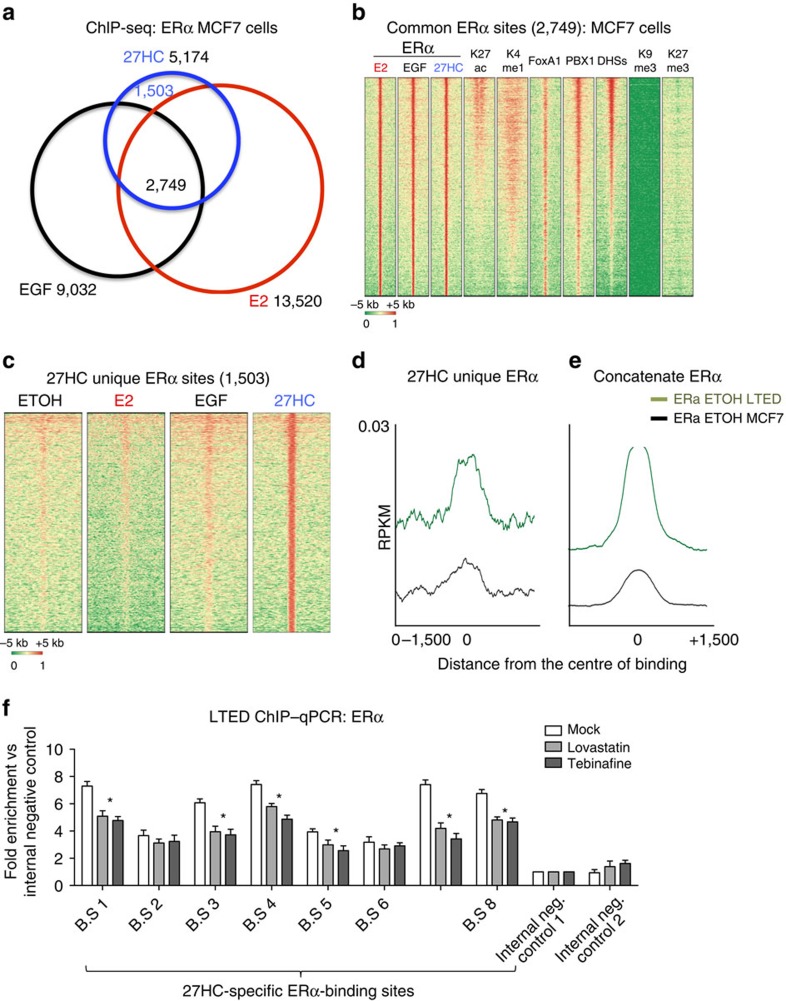
27-Hydroxycholesterol promotes ERα binding. (**a**) Venn diagrams of ligand-specific ERα cistromes. (**b**) Heatmaps showing the epigenetic features of ERα-binding sites common to all stimuli. (**c**) Heatmaps showing ERα binding at 27HC-specific loci. (**d**) Comparison between ERα ChIP-seq signals from LTED (green) and MCF7 (black) grown in the absence of estrogens at 27HC-specific sites. (**e**) Same analysis as in **d** focusing on all ERα-binding sites (right panel). (**e**) ChIP–qPCR analysis shows reduced ERα binding at 27HC-specific ERα-binding sites upon treatment with CB pathway inhibitors (48 h treatment). Error bars represent s.e.m. calculated on three independent experiments. Asterisks indicate significant differences compared with mock-treated cells (analysis of variance, with Dunnet's *t*-test *P*<0.05).

Next, we performed ChIP-seq analysis to determine whether 27HC could induce ERα chromatin binding in a way similar to other well-characterized ligands such as estradiol (E2)^38^ and EGF^40^. We performed these experiments using parental MCF7, in which endogenous CB is modest compared with LTED, to increase the signal to noise ratio and get robust ERα induction. 27HC stimulation (1 μM)^38^ led to ERα binding to 5,174 regions. Interestingly, over 70% of 27HC-ERα-binding sites were occupied by E2-ERα or EGF-ERα (Fig. 5a). Shared ERα-binding sites were enriched for H3K27Ac, H3K4me1, PBX1 and FOXA1, two TFs central to ERα signalling^19^ and were highly accessible (as shown by DHS-seq) while depleted of repressive histone marks (K9me3 and K27me3) (Fig. 5b). Shared ERα-binding sites had over 70% overlap with the core-ERα-binding sites found *in vivo*^41^. We thus concluded that 27HC-bound ERα can be recruited to regulatory regions commonly associated with oestrogen signalling. In addition, we also identified a set of binding sites unique to 27HC activation (Fig. 5c). These sites appeared to be weaker and less enriched for K27ac but still under selective constraints (Phastcons method).

We then examined ERα recruitment in LTED cells cultured in the complete absence of oestrogen. We hypothesized that endogenous CB activity might be sufficient to promote ERα binding to a large set of regulatory elements. Focusing on 27HC-specific ERα-binding sites, we found that oestrogen-depleted LTED cells have a higher average ERα binding compared with MCF7 cells grown in the same conditions (Fig. 5d). When we expanded this analysis to the entire repertoire of ERα-binding sites (E2, EGF and 27HC in MCF7 plus LTED, total 51,974 regions), LTED cells still exhibited stronger genome-wide recruitment of ERα compared with their parental MCF7 cells grown in the absence of oestrogen (Fig. 5e), suggesting that CB activation might be sufficient to replace oestrogen in LTED cells. Blocking endogenous CB using Lovastatin (HMGCR inhibitor) or Terbinafine (SQLE inhibitor) induces a modest although significant reduction in ERα recruitment at 27HC unique sites in LTED cells (Fig. 5f), further suggesting that CB contributes to cell-autonomous ERα recruitment in LTED cells.

**Figure 6:**
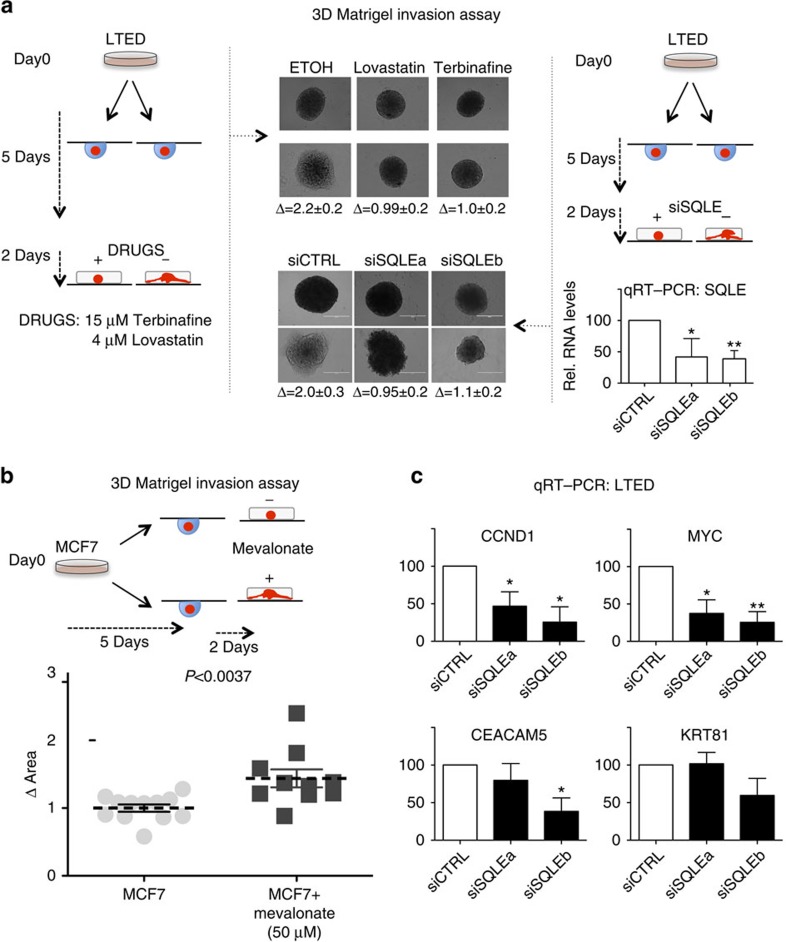
Blocking cholesterol biosynthesis reduces cellular invasion. (**a**) Blocking cholesterol biosynthesis via treatment with CB inhibitors (left panel) or direct depletion of SQLE (right panel) is sufficient to arrest LTED invasion in Matrigel organoids assays. The horizontal white bar indicate a distance of 400 μm. Δ Area is calculated using ImageJ (area day 2/area day 0, *n*=5). SQLE RNA fold changes were determined comparing two independent short interfering RNA (siRNA) (**a**,**b**) versus a non-targeting control. Bars represent the mean with the s.d. of three independent experiments. Asterisks indicate significant difference (analysis of variance (ANOVA), *P*<0.05). (**b**) Treatment with Mevalonate increases the invasive potential of MCF7 cells in 3D organoid assays. Δ Area is calculated using ImageJ (area day 2/area day 0). *P* value was calculated using a Student's *T*-test. (**c**) qRT–PCR analysis of genes involved in invasion/migration in LTED cells upon SQLE depletion. KRT81 is shown as a control. RNA fold changes were determined comparing two independent siRNAs versus a non-targeting control. Error bars represent s.e.m. calculated on three independent experiments. Asterisks indicate significant differences (ANOVA, *P*<0.05).

Finally, we reasoned that epigenetic CB activation in AI-resistant LTED cells might contribute to the invasive phenotype displayed by these cells. We therefore tested the possibility of blocking cellular invasion by targeting CB. As expected, LTED cells tended to be more sensitive to inhibition of CB as demonstrated by their respective half-maximal inhibitory concentration (IC_50_) (MCF7: 89 and 90 μM, LTED: 22 and 42 μM, Lovastatin and Terbinafine, respectively). More importantly, inhibition of CB or short interfering RNA-mediated depletion of SQLE was sufficient to block the invasion of AI-resistant LTED cells in 3D Matrigel assay (Fig. 6a; Supplementary Movie 5A–D). Conversely, treatment with mevalonate (the byproduct of HMGCR) significantly increased the invasive potential of non-invasive MCF7 cells (Fig. 6b). SQLE depletion also resulted in the significant repression of several genes involved in cellular invasion (Fig. 6c). Altogether, these data strongly suggest that epigenetically activated CB contributes to the invasive phenotype exhibited by AI-resistant LTED cells.

### CB serves as a biomarker in AI-treated breast cancer patients

Our integrative analysis of breast cancer cell lines resistant to individual ETs identified epigenetic activation of CB as a potentially important resistance mechanism specific to AI-treated patients. We therefore investigated whether our findings had translational potential and could be used to stratify patients before endocrine treatment. We initially developed a gene signature (Supplementary Data 1) to retrospectively stratify TCGA ERα-positive breast cancer patients that expressed high or low levels of CB gene mRNA levels. We used TCGA data since it was possible to retrieve treatment information from these patients (AI *n*=144, Tamoxifen *n*=127). ERα-positive patients with high expression of our CB-based signature at diagnosis consistently display shorter recurrence- and metastatic-free survival and are characterized by poor survival (Supplementary Fig. 8A,B). The same signature cannot be used to identify ERα-negative patients, suggesting a significant degree of specificity (Supplementary Fig. 8C). There are no significant changes in performance when correcting for luminal A or luminal B (Supplementary Fig. 9). CB can be directly upregulated in response to p53 mutations^42^; however, MCF7 cells are p53^wt^ and LTED do not acquire p53 mutations. On the other hand, T47-LTED incur in significant CB activation despite T47D being already p53^mut^, suggesting a degree of independence between the genetic status of p53 and the activation of CB. Finally, we still observed a strong prognostic significance within the ERα-p53-mutated subpopulation, suggesting the existence of additional mechanisms (Supplementary Fig. 9).

**Figure 7:**
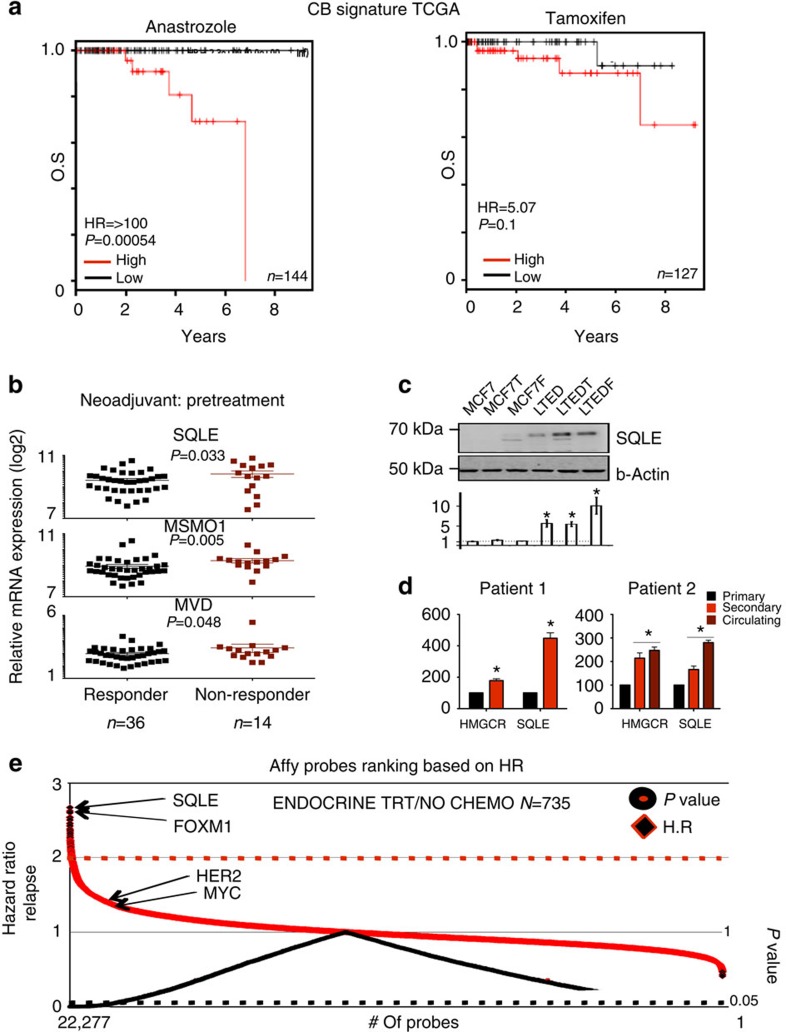
CB-guided stratification of AI-resistant patients. (**a**) Kaplan–Meier survival plots using averaged expression of differentially regulated enzymes (RNA-seq) involved in cholesterol biosynthesis in specific subgroups of ET-treated patients. (**b**) Pretreatment mRNA levels (FEMARA Trial, see text) of key enzymes can help identify AI-resistant patients. *P* values were calculated using a Mann–Whitney *U*–test. (**c**) Western blot analysis of SQLE and protein quantification. Error bars represent s.e.m. calculated on four independent experiments. Asterisks indicate significant difference comparing each cell type with MCF7 cells (analysis of variance, *P*<0.05). (**d**) HMGCR and SQLE mRNA levels were quantified in longitudinal samples from breast cancer patients. mRNA was extracted from FFPE slides marked by a registered pathologist. Error bars represent s.e.m. calculated on three independent experiments. Asterisks indicate significant differences compared with primary samples (*T*-test, *P*<0.05). (**e**) Manhattan plot for the prognostic potential of all human genes contained in the affy-chip U133. Both hazard ratio and *P* values are represented. Red and black dotted lines represent hazard ratio=1 and *P*<0.05, respectively.

Most importantly, our CB signature was particularly efficient in assessing overall survival in AI-treated patients but not in Tamoxifen-treated patients (Fig. 7a) again suggesting that endocrine resistance is driven by distinct mechanisms. Examining the expression of genes involved in CB in an independent cohort (FEMARA trial^43^), we find that MSMO1, MVD and SQLE mRNA levels are statistically higher in patients that did not respond to neo-adjuvant (pre-surgery) AI therapy (Fig. 7b). Overall, these data suggest that patients with high CB are less likely to benefit from AI treatment and are in substantial agreement with our *in vitro* results.

SQLE is the second rate-limiting enzyme in the CB pathway and one of the genes epigenetically activated specifically in LTED cells (Figs 2g and 4b). As expected, SQLE-encoded protein is strongly upregulated in all LTED models (Fig. 7c). We therefore quantified mRNA levels for HMGCR and SQLE in an ERα breast cancer patient treated with AI for whom we had longitudinal data (RNA obtained at diagnosis, first relapse and in circulating metastatic cells from pleural effusion). In agreement with our previous finding, we found that key CB genes are progressively upregulated during cancer progression (Fig. 7d). Overall, SQLE emerged from these analyses as a novel candidate biomarker to identify AI-resistant breast cancer patients. This was further confirmed by our unbiased analysis of the entire human transcriptome. When we sorted all 22,277 probes present in the U133A gene chip according to their potential to predict relapse in a panel of 724 ERα-positive breast cancer patients treated with ET, SQLE (probe set 209218_at) ranked 1st (hazard ratio=2.67) (Fig. 7e; Supplementary Data 5). Finally, we speculated that genetic–epigenetic interactions might facilitate SQLE expression in ERα-positive breast cancer. Remarkably, SQLE is amplified in almost 10% of ERα-positive patients and overexpressed in an additional 13%^44^ (Supplementary Fig. 10A). SQLE amplification directly correlates with increased mRNA expression in primary tumours (Supplementary Fig. 10B). SQLE emerges also as one of the top-ranking overexpressed genes in 10 breast cancer-independent data sets (invasive ductal carcinoma (IDC) versus normal breast, Supplementary Data 6). More importantly, SQLE amplification or mRNA overexpression is sufficient to stratify outcome in ERα-positive breast cancer patients from the TCGA cohort (Supplementary Fig. 10C). Altogether, our data strongly support the prediction generated using drug-specific resistant cells and confirm a functional role for epigenetic activation of CB as a mechanism of resistance to AI therapy *in vivo.*

## Discussion

In addition to clonal selection^41^, adaptation to drug treatment may involve heritable transcriptional changes that allows cells to survive in the presence of externally imposed selective pressure^7^,^8^. Using ERα-dependent breast cancer cells as a working model, we demonstrate that adaptation to therapeutic agents sharing the same target follow distinct routes depending on the specific mechanism of action. More specifically, the appearance of oestrogen-independent growth corresponds to the epigenetic activation of CB. CB functionally contributes to the invasive phenotype AI-resistant cells and CB activation can be used to predict resistance to AI before treatment. Drug-resistance, however, does not invariably coincide with the development of more aggressive phenotypes, suggesting that in some instances the transition to metastatic progression requires additional reprogramming. Using recently developed paradigms, our data demonstrate that individual drugs impose very specific epigenetic changes that target both local regulatory elements such as individual enhancers and large topological domains and SEs^11^. This is surprising considering that endocrine treatments impinge on the same TF (for example, ERα). It will be important to evaluate whether a similar process occurs also in other malignancies.

TF motif analyses of epigenetically reprogrammed loci show that deregulation of the core network of TFs, which lies at the basis of cell-type specification (for example, FoxA1)^45^ might lead to aberrant rearrangements of entire chromosomal territories. These rearrangements may include surrounding genes that are central to functional reprogramming, drug resistance and invasion. At the same time, specific selective pressures might trigger epigenetic activation of tailored pathways (for example, CB), irrespective of the broad chromatin context in which single genes are nested.

Our data support a model in which chronic exposure to AI constitutively activates CB. Intra-tumour activation of CB may then lead to the local accumulation of metabolic ligands for other nuclear receptors. Promisingly, we find that AI-resistant SEs are enriched with DNA motifs of metabolic nuclear receptors including FXR and LXR. It is well established that 27HC is also an LXR agonist^38^, therefore we cannot exclude that LXR might also contribute to AI resistance in combination with endogenous ERα activation^46^. In addition, endogenously produced ligands, including 27HC might act on additional nuclear receptors and allow the activation of an alternative cohort of regulatory elements (Supplementary Fig. 11). Of note, 40/48 nuclear receptors (NRs) have increased expression in LTED cells (FXR>300 fold, LXR>1.8 fold) and 9 of these are markedly increased in ERα-negative LTEDF (PPARγ> 500-fold) strongly suggesting that other nuclear receptors might contribute to the invasive phenotype observed in LTEDF cells. In the same way, autocrine metabolic signalling might also contribute to ERα binding found in metastatic breast cancer *in vivo*^41^.

Epigenetic activation of the CB pathway might also be supported by specific genetic aberrations. For example, SQLE amplification occurs in a significant proportion of cancers^47^, but the functional consequences of SQLE amplification have not previously been appreciated. SQLE locus is just 2 Mb away from the oncogene *Myc* in a hotspot for genomic rearrangements^44^. However, we could not find any clear correlation between SQLE and MYC expression, suggesting that the poor outcome typical of breast cancer patients with high SQLE does not simply reflect MYC activation. Notably, our model could partially explain recently published epidemiological observations. While the protective role of anti-cholesterol statin towards breast cancer is still debated, a nation-wide study found that statin users with ERα-positive breast cancer are less likely to develop relapses^48^. In agreement with our findings, no effect on relapse rates was noted in patients with ERα negative^48^

Recent clinical trials recommend a switch from Tamoxifen to AI with ovarian suppression in pre-menopausal women^49^. Our data suggest that Tamoxifen and AI exposure do not have an equivalent effect on breast cancer cells; thus ERα patients should be assigned to a specific endocrine treatment depending on the molecular profile of their tumour. Moreover, our data offer novel molecular insights and warrants for additional clinical trials combining statins and AI in patients with metastatic disease^47^. Pharmacodynamics studies show no influence of statins on E2 levels or Anastrozole metabolism^50^. Interestingly, statins were shown to interfere with ovarian cancer and multiple myeloma growth in pre-clinical models^51^,^52^. Our work therefore offers a new paradigm in the context of how epigenomes evolve in response to interrelated exogenous pressures while paving the way for future studies designed to help us understand the specific effects of therapeutic intervention during metastatic progression.

## Methods

### Cell lines and clinical samples

Cell lines were cultured as follows MCF7 and T47D: DMEM cell growth medium (DMEM with 10% of fetal bovine serum (FBS) and 100 U penicillin/0.1 mg ml^−1^ streptomycin plus 10^−8^ 17-β-estradiol (SIGMA E8875). LTED cells were passaged in stripped DMEM cell growth medium (Phenol-free DMEM with 10% of charcoal-stripped FBS (CDT) and 100 U penicillin/0.1 mg ml^−1^ streptomycin MCF7T and LTEDT were cultured as MCF7 and LTED with the addition of 10^−7^ 4-hydroxytamoxifen (SIGMA H7904). MCF7F and LTEDF were cultured as MCF7 with the addition of 10^−7^ Fulvestrant (SIGMA I4409)^15^. Tissues (primary, secondary and pleural effusions) were obtained from the Imperial Tissue Bank. The Tissue Management Committee reviewed and approved all documents. Imperial Tissue bank released stores the samples under an HTA Licence (12275). The appropriate regulatory committees approved all the relevant documents. Each patient signed an informed consent to donate tissue to Imperial Tissue Bank. All patients' notes are kept within locked filing cabinets within locked offices in research. This is to maintain confidentiality. Tissues used for qRT–PCR were manually enriched for tumour cells by the resident pathologist. Pleural effusions were cultured in DMEM, 10% FBS and phenol for a brief time before RNA extraction. Cells were tested, genotyped and characterized as ERα positive (LGC standards). Circulating tumour cells were characterized as mammary gland based on short tandem repeat similarity with HTB-22 (LGC standards).

### Invasion assays

Boyden chamber and 3D Matrigel invasion assays were performed as previously described^53^. Briefly, cells (5 × 104 in 200 μl of α-MEM) were plated in the Matrigel-coated upper chambers of the 24-well Transwell invasion assay plate (Corning). Each condition was represented in triplicate. Plates were incubated at 37 °C for 24 h for HCC1806 cells. Cells in the lower chamber (including those attached to the under surface of the membrane) were trypsinized and counted using a Casy 1 counter (Sharfe System). Mean and s.e.m. of independent experiments were calculated. Statistical analysis was performed using a two-tailed Student's *t*-test to determine the statistical significance of the differences observed. A *P* value below 0.05 was considered significant.

### Fatostatin and mevalonate treatments

Cell lines were cultured in the appropriate conditions until 60–70% confluence and then treated with 15 μM of Fatostatin (SIGMA F8932) for 6 h before RNA extraction and qRT–PCR analysis. For mevalonate treatment, organoids were allowed to form and embedded in Matrigel (see Hanging-drop assay) then treated with 50 μM mevalonate^54^ (SIGMA 90469).

### Mouse models

MCF7-mCherry-Puro and LTED-mCherry-Puro cells (1 × 10^6^) were injected into the tail vein of NOD-SCID mice (*n*=3 per group). Images were taken of the mice weekly from dorsal and ventral views for 8 weeks to monitor the development of metastases. The mice were assessed weekly using whole-body imaging to quantify the relative amounts of tumour burden. Imaging was performed with a highly sensitive, cooled charge-coupled device camera mounted in a light-tight specimen box (IVIS; Xenogen). The imaging and quantification of signals were controlled by acquisition and analysis software. At termination, the lymph nodes, lungs, kidney, bones and livers were harvested by dissection. The lymph nodes, lungs, kidneys, liver and bones were fixed for 24 h in neutral buffered formalin, and then rinsed in 70% ethanol. Following fixation, the bones were decalcified in 10% EDTA in 0.1 M Tris-HCl pH 7.4 for 1 week, re-fixed in formalin and then rinsed in 70% ethanol. All tissues were dehydrated using ethanol, cleared with Histoclear and embedded in paraffin wax. Paraffin sections (5 μm) were stained with haematoxylin and eosin to reveal their tissue morphology and the presence of infiltrating cells. The animal trials were carried out under London Home Office license authority and London Home Office Ethics Committee guidelines.

### Immunohistochemistry and western blot

Paraffin sections were dewaxed and antigen retrieval was carried out with citrate buffer at pH 6.0 or in 10 mM Tris-HCl, pH 9.0. Tissue sections were pretreated using 0.3% H_2_O_2_ in PBS, rinsed in PBS and then incubated with 20 μl ml^−1^ normal goat serum. Primary antibodies were diluted in PBS and incubated overnight at 4 °C, along with negative control sections where the primary antibody was omitted. All the bound antibodies were detected using biotinylated anti-mouse or anti-rabbit secondary antibodies, detected using the Vectastain Elite peroxidase ABC kit and the ImmPACT DAB kit (Vector Laboratories). Stained sections were counterstained with haematoxylin. Antibody dilutions: PBX1 1:50 (Abnova H00005087-M01), Human nuclear antigen 1:25 (Abcam ab191181), Pan-cytokeratin 1:100 (SIGMA C5992). For western blot, SQLE antibody (SIGMA HPA018038) was used at 1:250 and ERα (Santa Cruz sc-543) was used at 1:1,000.

### Hanging-drop assay

Briefly, cells were trypsinized and counted. In all, 250,000 cells were resuspended in 1 ml of phenol-red or phenol-red-free DMEM with the relevant concentrations of Estradiol, Fulvestrant and Tamoxifen. Twenty-microlitre drops were dispensed on to a 10-cm dish lid. Five millilitre of medium were added to the bottom of the dish to prevent evaporation. The lid was inverted and the drops were left hanging for 5 days. The newly formed organoids were then transferred into 10 μl Matrigel drops (BD 356237, BD 356234) and moved to 24- or 48-well plates after which immunofluorescence or real-time monitoring (respectively) was carried out. Two hundred microlitre of medium with the relevant concentration of drugs were added to the wells.

### Real-time invasion and migration assays

Cell invasion and migration were assessed in real time using the xCELLigence system CIM-16 wells plates. The bottom chamber was filled with 160 μl of full medium (phenol-red or phenol-red-free DMEM with the relevant concentrations of Estradiol, Fulvestrant and Tamoxifen) and acted as a chemoattractant. The upper chamber was then secured on top and 30 μl of serum-free medium were added to avoid the membrane drying out. The plate was loaded on the machine cradle and left for 1 h to allow membrane equilibration. After 1 h, the baseline measurement was taken. Hundred microlitre of serum-free medium (again phenol-red or phenol-red-free DMEM with the relevant concentration of Estradiol, Fulvestrant and Tamoxifen) containing 40,000 cells were subsequently added to each well, and left for 30 min at room temperature to allow for the attachment of the cells. The impedance value for each well was recorded every 15 min for 20 h for migration experiments and 48 h for invasion and expressed as Cell Index value. For invasion, the CIM plates were coated with 30 μl of a 1:40 dilution of Matrigel (BD Biosciences) and allowed to set for 4 h at 37 °C before proceeding as per the migration experiment. Four wells per condition were used in each experiment and every experiment was repeated four times.

### Chromatin immunoprecipitation (ChIP)

Cells were crosslinked with 1% formaldehyde and processed according to Schmidt *et al.*^55^ Briefly, chromatin extract were sonicated using a Diagenode sonicator using 20 cycles (30 s on and 30 s off) at maximum intensity. Purified chromatin was then immunoprecipitated using 4 μg of K27ac antibodies (Abcam ab4729) per ChIP. Non-immunoprecipitated chromatin was used as Input control. Chromatin was then decrosslinked and sonication efficiencies tested on a 1.5% agarose gel. Before the construction of ChIP-seq libraries (NEB, see Supplementary Methods), we determined the enrichment using positive and negative controls. We then used 10 ng of IPed chromatin and 10 ng of Input for library preparation.

### Bioinformatic analyses

Detailed protocols on Peak Calling, enhancers-promoters Peak-pairing, Clustering analysis, IPA analysis and RNA-seq differential expression analysis can be found in the Supplementary Methods.

### RT–qPCR and ChIP–qPCR

RT–qPCR and ChIP–qPCR were performed and analysed as follows. Briefly, reactions were carried on in 10 μl volume containing 5 μl of SYBR mix (ABI 4472918), 0.5 μl of primers (2.5 and 5 μM final concentration, respectively), 2.5 μl of complementary DNA or DNA and 2 μl of water. We used a three-step cycle programme with melting analysis. The cycles were as follow: 10 s at 95 °C, 30 s at 60 °C and 30 s at 72 °C, repeated 40 times^7^.

### SRB and IC_50_ assay

SRB and IC_50_ assays were performed as follow: briefly, the sulphorhodamine B (SRB) assay was used to monitor the effects of statin or Terbinafine treatment cell on cell proliferation in monolayer cultures. Cells were seeded in flat-bottomed 96-well plates (3 × 10^3^ cells per well). The cells were allowed to adhere overnight. One plate was assayed at this time point (day 0) and further plates were assayed at 2-day intervals. The cells were fixed by adding 100 μl per well of ice-cold 40% (vol/vol) TCA to each well for 60 min. The plates were washed five times in running tap water and stained with 100 μl per well of the SRB reagent (0.4% wt/vol SRB in 1% (vol/vol) acetic acid) for 30 min. The plates were washed five times in 1% (vol/vol) acetic acid and allowed to dry overnight. SRB was solubilized with 100 μl per well 10 mM Tris-base, shaken for 30 min and the optical density was measured at 492 nm.

### Primers

The sequences of primers (RT–qPCR, ChIP–qPCR) are listed in Supplementary Data 7.

## Additional information

**Accession codes:** RNA-seq can be accessed at ENA (http://www.ebi.ac.uk/ena) with the code PRJEB11448. ChIP-seq data can be found in the GEO archive under number GSE60517.

**How to cite this article:** Nguyen, V. T. M. *et al.* Differential epigenetic reprogramming in response to specific endocrine therapies promotes cholesterol biosynthesis and cellular invasion. *Nat. Commun.* 6:10044 doi: 10.1038/ncomms10044 (2015).

## Supplementary Material

Supplementary Information Supplementary Figures 1-11, Supplementary Methods and Supplementary References.

Supplementary Movie 1 MCF7 cells invasion assay.

Supplementary Movie 2 MCF7T cells invasion assay.

Supplementary Movie 3 MCF7F cells invasion assay.

Supplementary Movie 4 LTED cells invasion assay.

Supplementary Movie 5 Mock treated LTED cells invasion assay.

Supplementary Movie 6 Ethanol treated LTED cells invasion assay.

Supplementary Movie 7 Lovastatin treated LTED cells invasion assay.

Supplementary Movie 8 Terbinafine treated LTED cells invasion assay.

Supplementary Data 1 Ingenuity Pathway analysis for upregulated genes in ETR cells. We compiled the total gene list for upregulated genes (all ETR vs. MCF7) and then created gene sets that were found expressed at higher levels in only one cell type (by excluding genes found in more than one cell type). Next we combined MCF7T and MCF7F vs. LTED, LTEDT and LTEDF and ran IPA. IPA canonical pathways, Upstream regulator analysis, Biological function analysis and Network analysis are reported in sub-tables.

Supplementary Data 2 ETR-specific prognostic gene signatures. We obtained ETR-specific gene signatures by collecting the top 10% of upregulated genes in MCF7 vs. ETR using RNA-seq data. Genes can be contained in more than one list and the gene number is different since the number of differentially regulated genes is considerably different depending on the cell types. The overlap between different gene signatures is extremely limited, especially between MCF7T/MCF7F vs. LTED/LTEDT/LTEDF.

Supplementary Data 3 IPA analysis of epigenetically reprogrammed regions. We run IPA pathways using the clusters (13) identified using dynamically reprogrammed H3K27ac regions. Enrichment analysis are reported for each cluster using a -Log10 (p-value) (Fisher's exact test) along with their ratio (the number of total genes in the pathway found de-regulated divided by the total number of genes annotated in the pathway). The list of genes in the pathway is also provided in the last column.

Supplementary Data 4 Cell type specific expression of SE clusters-associated genes. Considering the genes putatively regulated by the SE-regions identified, FPKM values from RNA-seq are provided (separately for each cluster).FPKM values are reported for each individual cell line.

Supplementary Data 5 Single gene prognostic classifiers. Ranked list of all probes contained in the U133A, affymetrix microarray according to their respective Hazard Ratios calculated in a subset of breast cancer patient (ERa) treated with endocrine therapies.

Supplementary Data 6 SQLE mRNA change of expression comparing normal vs. breast cancer. 10 independent datasets were analyzed to evaluate SQLE expression in normal and invasive ductal carcinoma. Imperial College of Science, Technology and Medicine Individual datasets are labelled according to the original publication. Analysis were performed using Oncomine2.

Supplementary Data 7 List of primers used in the current study.
