# Author Correction: Differential epigenetic reprogramming in response to specific endocrine therapies promotes cholesterol biosynthesis and cellular invasion

**DOI:** 10.1038/s41467-019-11569-z

**Published:** 2019-08-06

**Authors:** Van T. M. Nguyen, Iros Barozzi, Monica Faronato, Ylenia Lombardo, Jennifer H. Steel, Naina Patel, Philippa Darbre, Leandro Castellano, Balázs Győrffy, Laura Woodley, Alba Rodriguez-Meira, Darren K. Patten, Valentina Vircillo, Manikandan Periyasamy, Simak Ali, Gianmaria Frige, Saverio Minucci, R. Charles Coombes, Luca Magnani

**Affiliations:** 1 Department of Surgery and Cancer, Imperial College London, London, W12 0NN UK; 2 IFOM-IEO Campus, European Institute of Oncology, Milan, 20139 Italy; 3 School of Biological Science, University of Reading, Reading, RG6 6LA UK; 4 MTATTK Lendület Cancer Biomarker Research Group, 2nd Department of Pediatrics, Semmelweis University, Budapest, H-1117 Hungary; 5 MTA-SE Pediatrics and Nephrology Research Group, 2nd Department of Pediatrics, Semmelweis University, Budapest, H-1117 Hungary; 6 ECMC, Charing Cross Hospital, London, W120nn UK; 7 Department of Pharmacy, Health and Nutritional Sciences, University of Calabria, Arcavacata di Rende (CS), 87036 Italy

Correction to: *Nature Communications* 10.1038/ncomms10044, published online 27 November 2015

This Article omits a declaration from the Competing Interests statement, which should have included the following: ‘One of the authors, Y.L., is an editor on the staff of *Nature Communications*, but was not in any way involved in the journal review process. The other authors declare no competing interests.’

Also, the Article contains an error in the name of the author Alba Rodriguez-Meira, which is incorrectly given as Alba Meira.

These errors have not been corrected in either the PDF or HTML versions of the Article.

