# Supplementary material for: Differential epigenetic reprogramming in response to specific endocrine therapies promotes cholesterol biosynthesis and cellular invasion
Source: Nat Commun. 2015 Nov 27;6:10044. doi: 10.1038/ncomms10044 (PMC4674692; doi:10.1038/ncomms10044)
Supplement: Supplementary Information — Supplementary Figures 1-11, Supplementary Methods and Supplementary References. [file ncomms10044-s1.pdf]

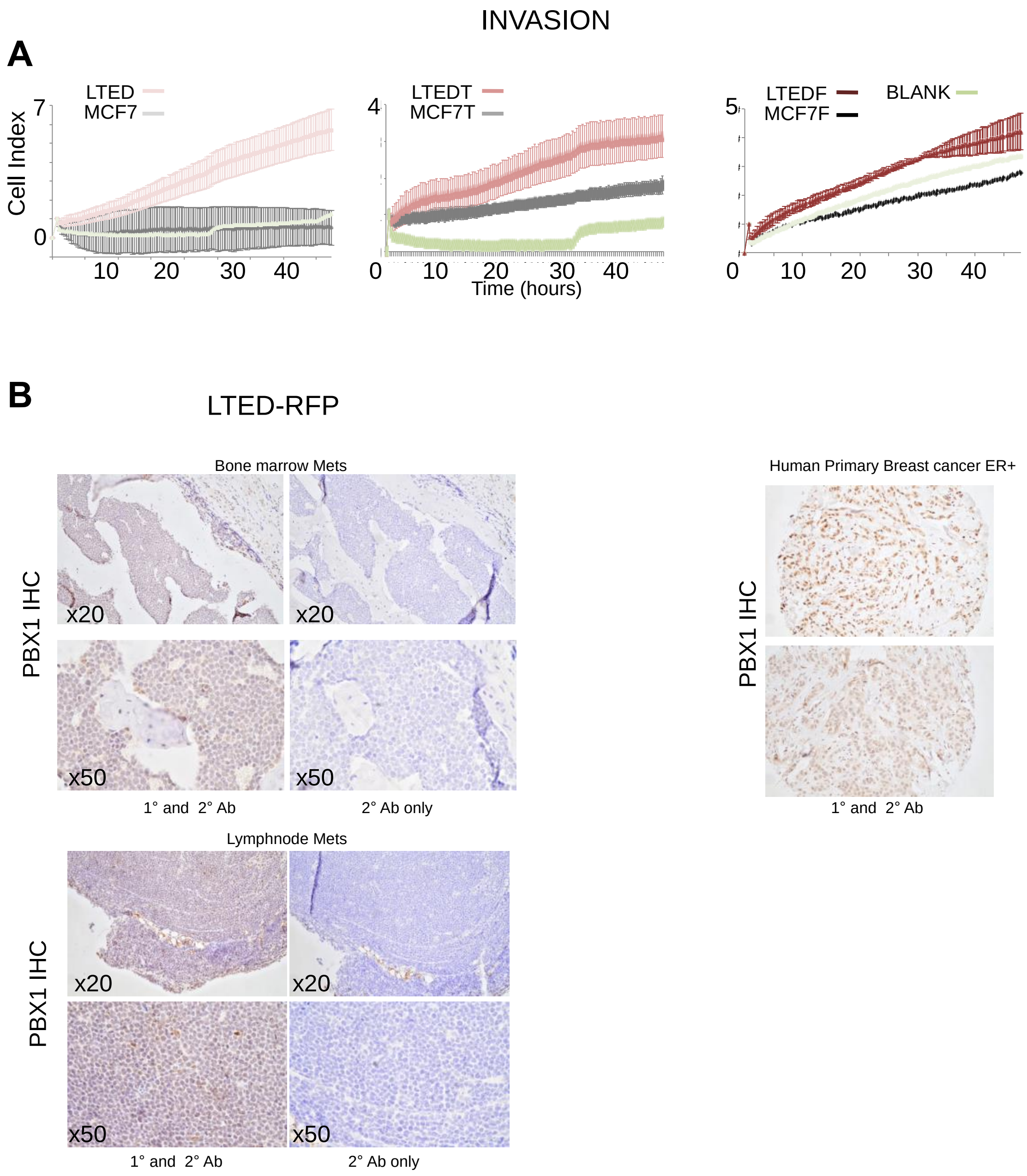

Supplementary Figure 1 A) Real-Time monitoring of invasion demonstrates the increased motility of LTEDs cells compared to other resistant lines. B) Pathological staining using human-specific antibodies that target a breast-cancer-specific pioneer factor[1]. The right-hand panel contains human-derived breast cancer primary tumours.

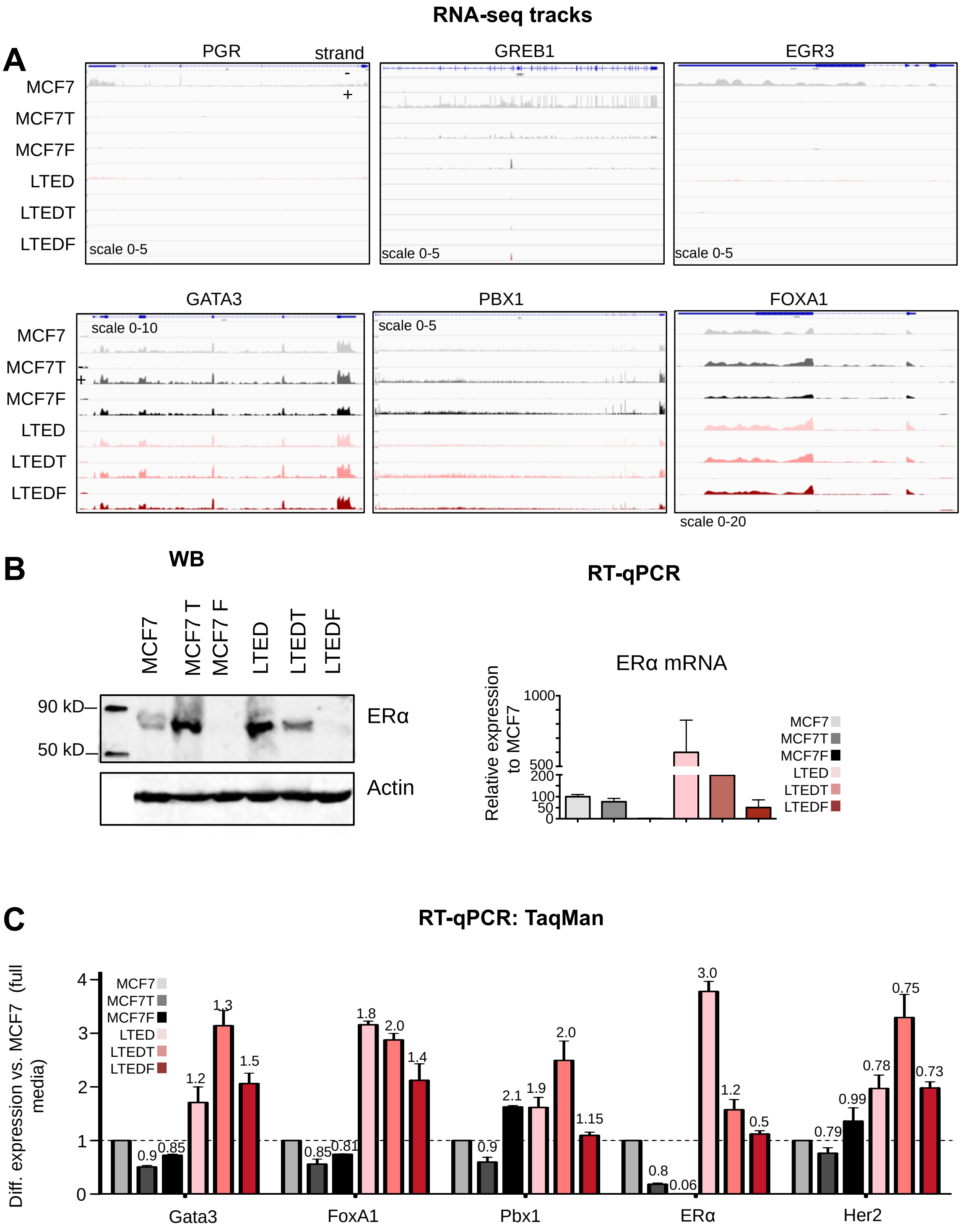

Supplementary Figure 2 A) Representative RNA-seq track of ERα target genes and ERα-associate pioneer factors. B) Western Blot analysis and expression analysis for ERα. Error bars represent SEM calculated on three independent experiments. C) TaqMan assay

on ERα signalling components in all cell lines cultured in full media (MCF7). The number at the top of the columns represents fold changes derived from RNA-seq analysis. Error bars represent SEM calculated on three independent experiments.

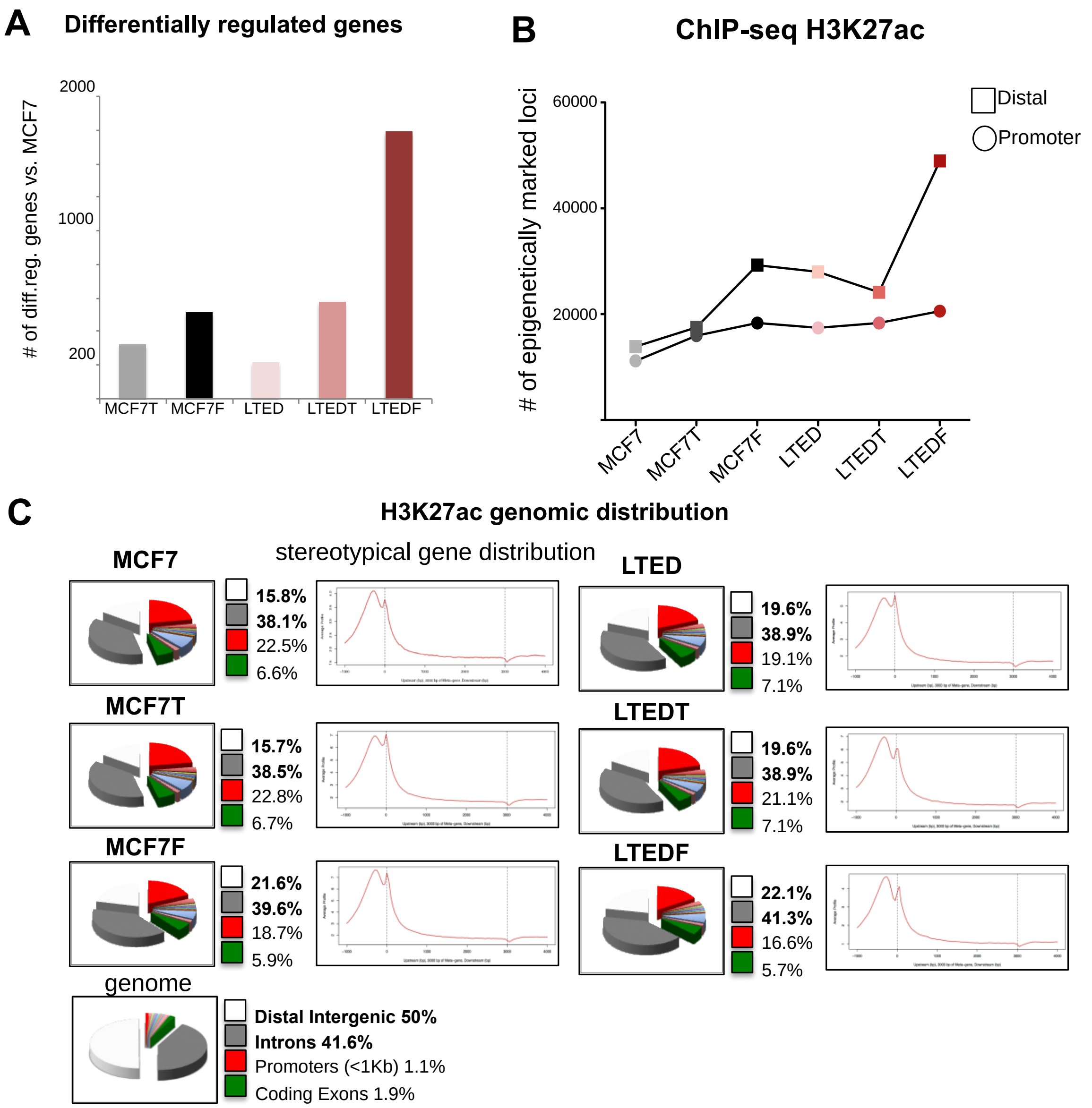

Supplementary Figure 3 A) Number of differentially regulated genes in ETR cell lines compared to MCF7 B) Total number of putative regulatory regions active in individual cell lines C) The stereotypical gene profiles and genomic distribution of H3K27ac regions identified in the six cell lines

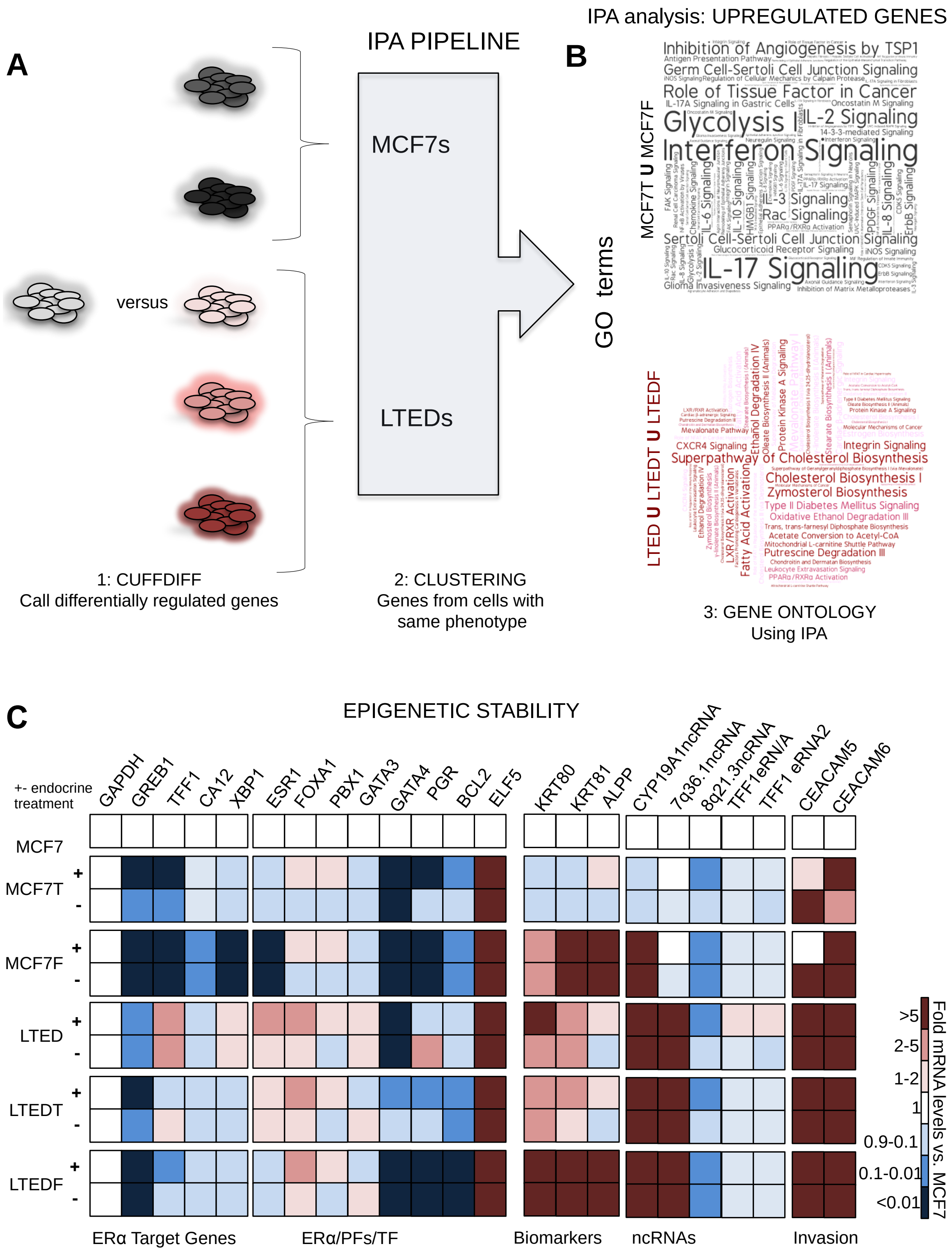

Supplementary Figure 4 A) Clustering strategy for RNA-seq based signatures. B) RT-qPCR analysis of differentially regulated genes in the presence (+) or absence (-) (full

media, MCF7) of drugs. Changes in gene expression compared to MCF7 (in full media) are shown as a heatmap.

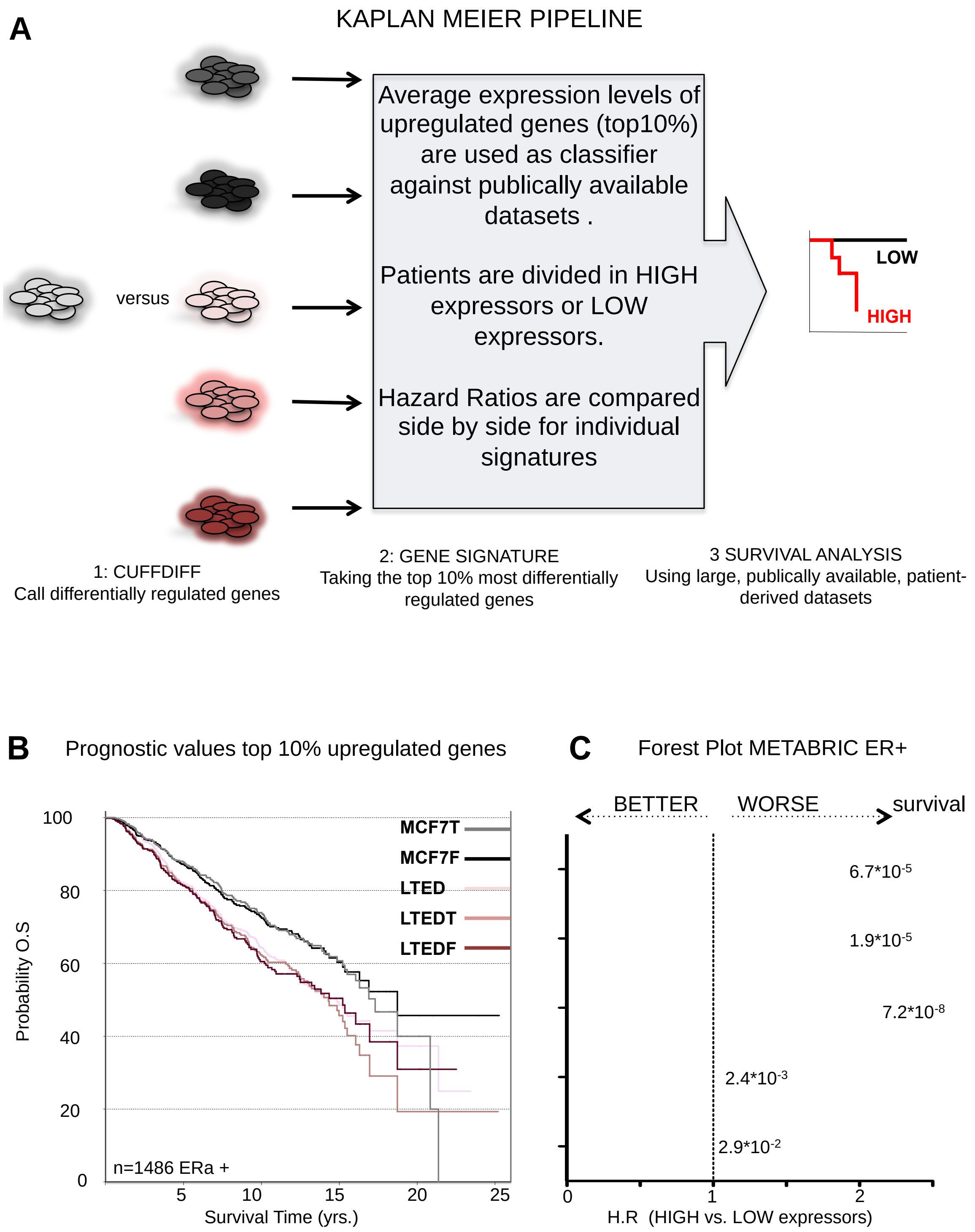

Supplementary Figure 5 A) Pipeline for Kaplan-Meier stratification of clinically derived transcriptional datasets. B) Kaplan-Meier combined plots of patients expressing high levels of genes up-regulated in individual resistant cell lines (the curves of low-

expressers are omitted for clarity). C) Forest plots of HRs for all cell-type-specific gene signatures. HRs and P values are calculated comparing Metabric ER+ patients expressing High vs. Low RNA levels for the individual signatures (as in panel C). The gene identifiers for each signature are in Table S2.

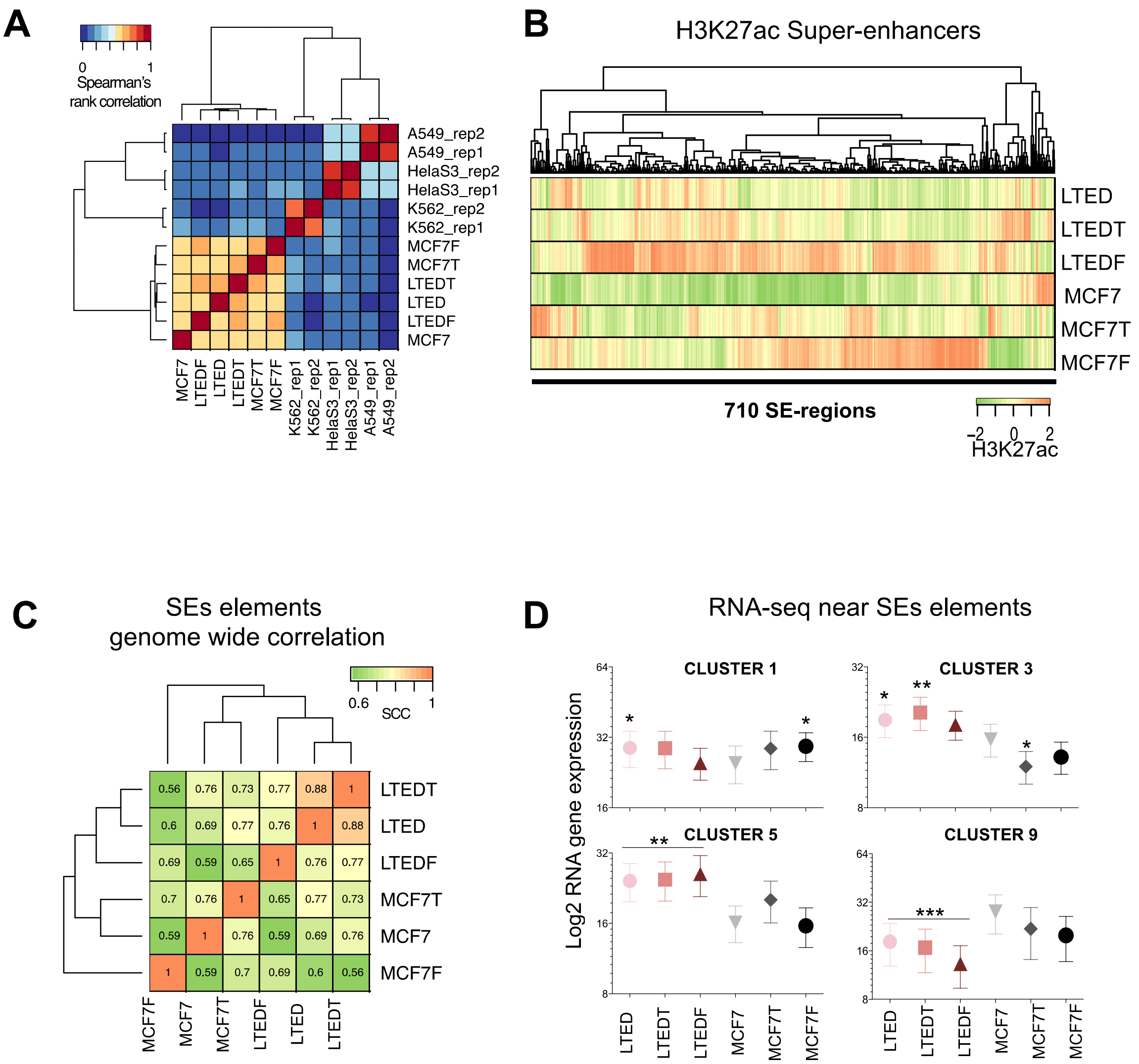

Supplementary Figure 6 A) Heatmap of spearman's correlation values comparing H3K27ac regions in ETR cells lines and other ENCODE cancer lines (leukaemia's K562, cervical carcinoma's HelaS3 and lung cancer's A549). B) Regions identified as SEs in at least one cell line were clustered by Pearson's correlation and shown as a heatmap. C) Correlation matrix of all six cell lines using SEs K27ac levels. D) Log2-ratio of mRNA levels (RNA-seq) for genes physically located near individual SEs clusters (1-3-5-9) show cell-type-specific transcription. Asterisks represent significance level (ANOVA *=<0.05, **=<0.01, ***<=0.001).

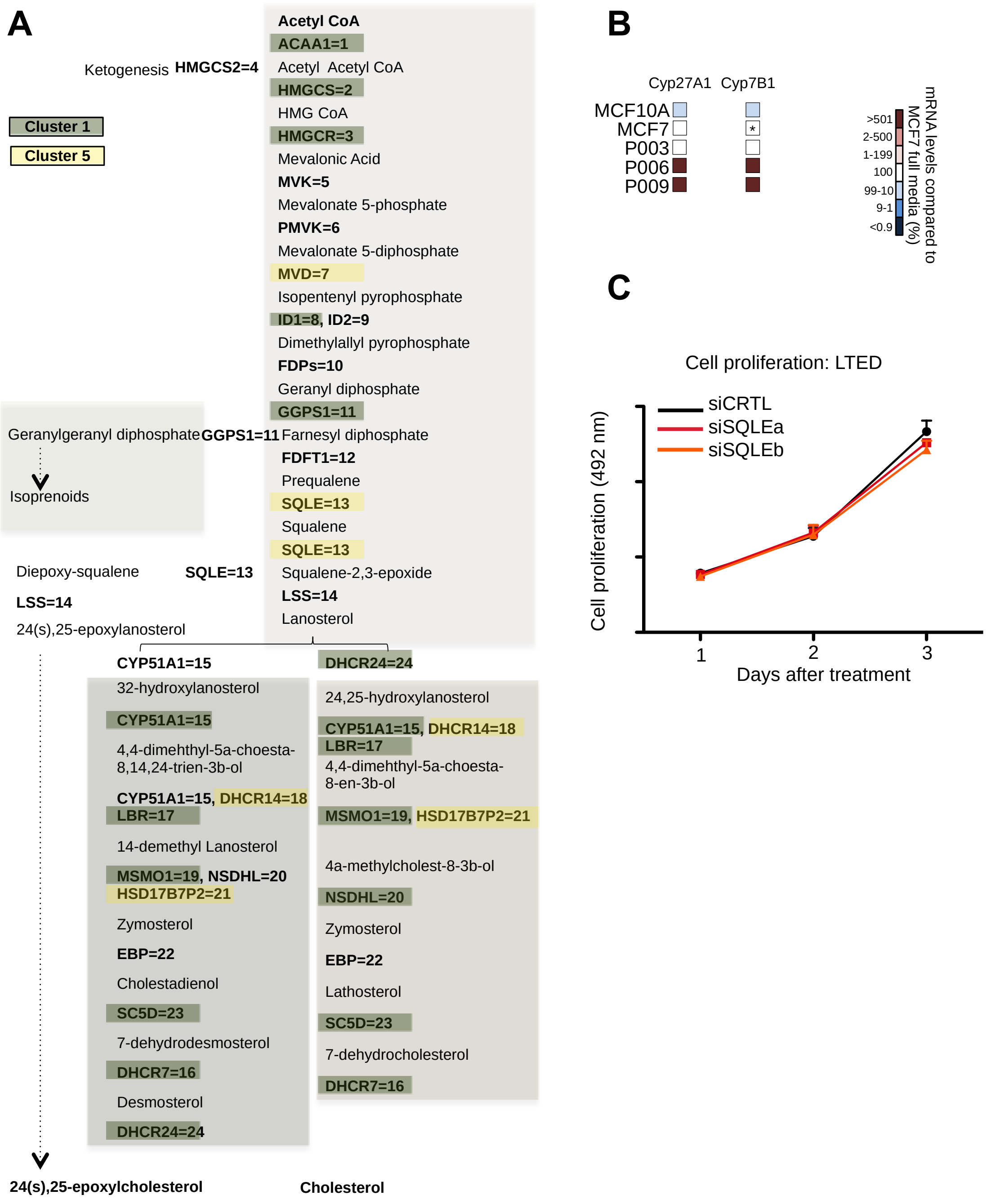

Supplementary Figure 7 A) Visual representation of the cholesterol biosynthesis pathway and associated primer numbers (as in fig. 4). Coloured boxes refer to epigenomic clustering. B) RT-qPCR analysis of the enzymes involved in 27HC

biosynthesis and catabolism. RNA fold differences were calculated comparing MCF7 to epithelial non-tumorigenic- (MCF10A) and in vivo derived resistant-cells (purified pleural effusion from 3 independent patients) averaging at least three independent experiments. C) Cell proliferation assay for cells treated with siRNAs against SQLE. The data represents averages and SEM from three independent experiments.

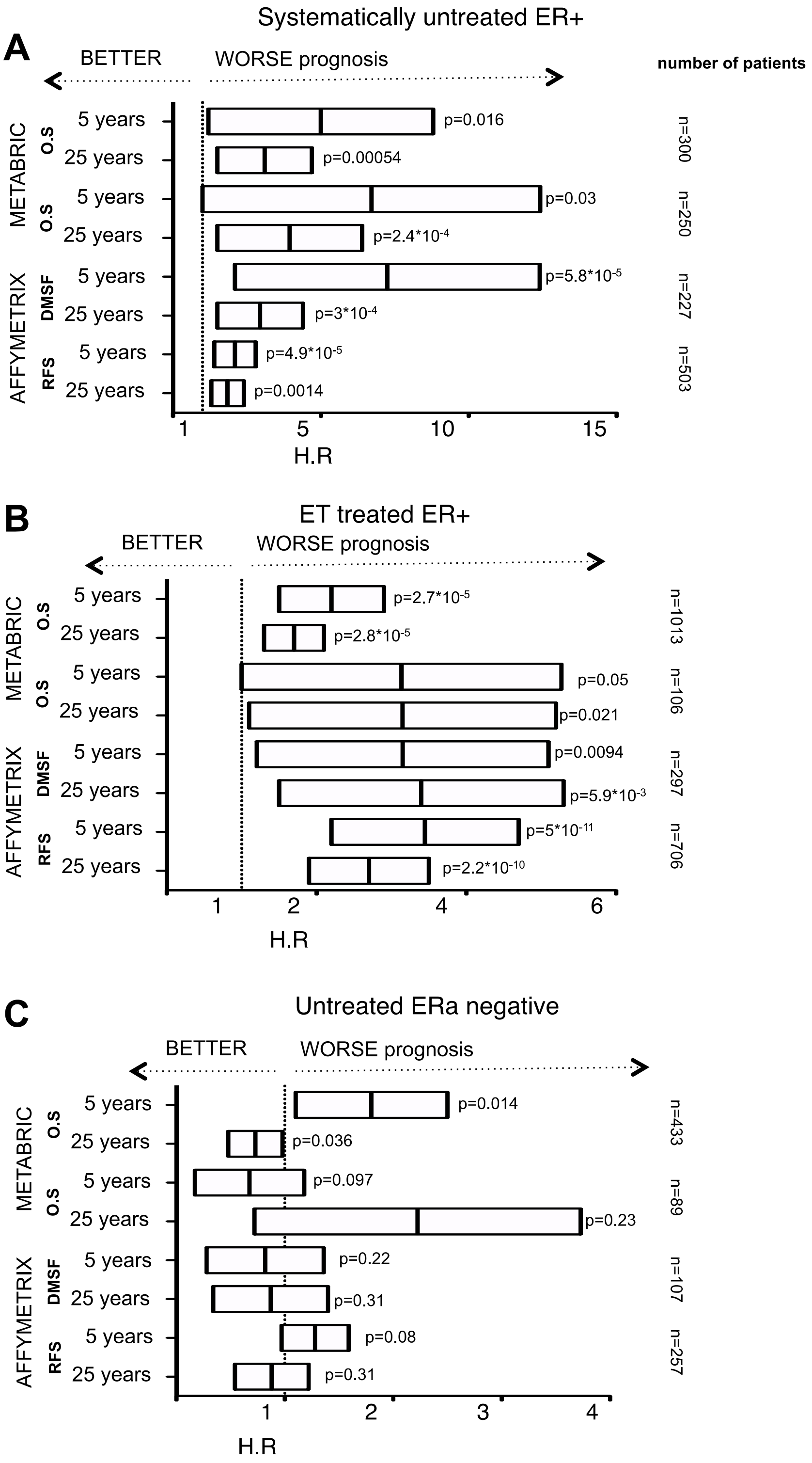

Supplementary Figure 8 A) Forest plot for the cholesterol-based signature shows prognostic potential in several publically available datasets. Patients were stratified in several subgroups based on treatment and ERα status: untreated (A), treated with

endocrine therapies (B), untreated ERα negative (C). The number of patients for each analysis is indicated in brackets. P values are indicated next to the confidence interval boxes.

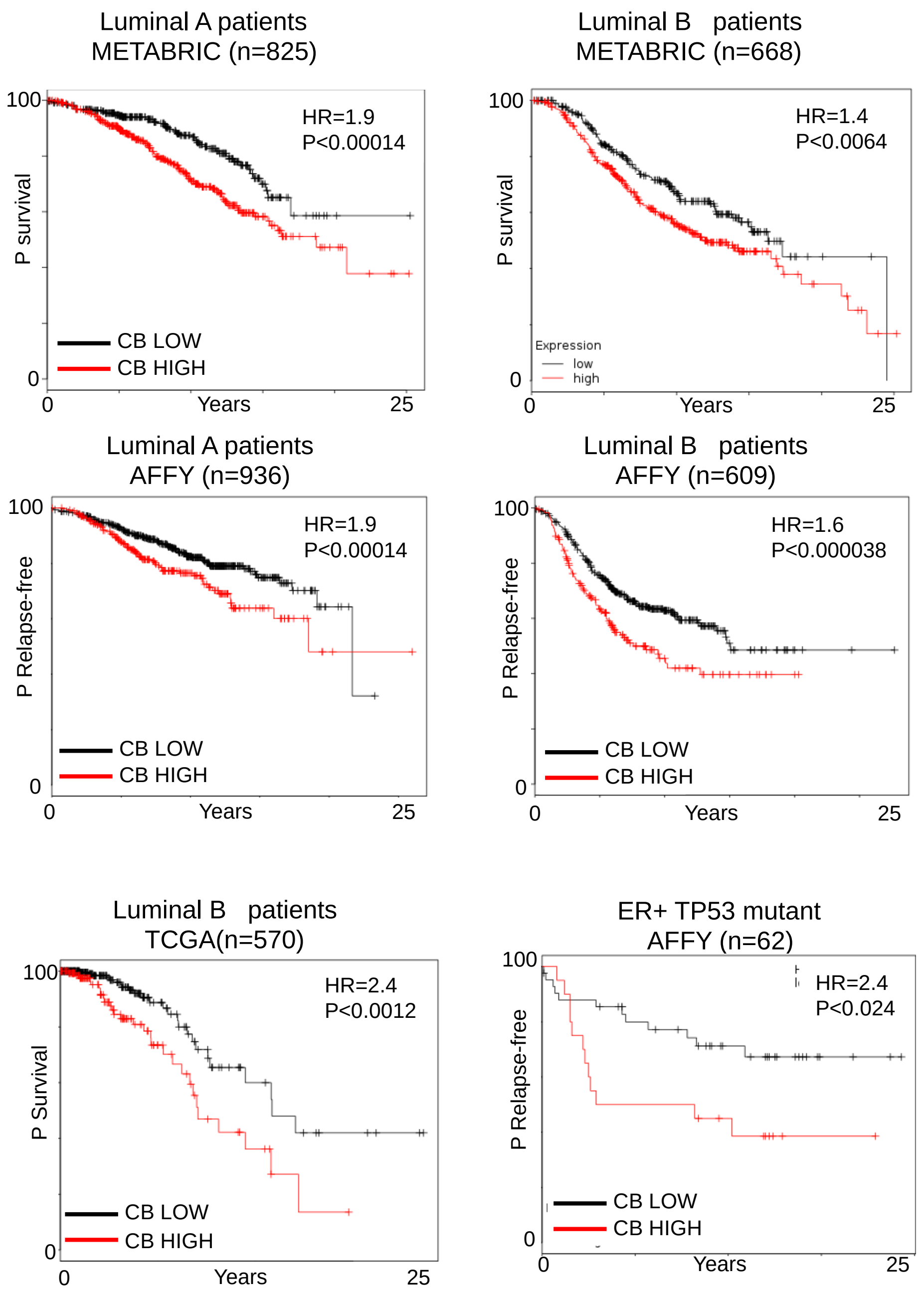

Supplementary Figure 9 Kaplan-Meier plots of luminal A and luminal B patients for overall- and relapse-free survival analysis. The patients were stratified using the same CB gene expression signature as in Fig. S8.

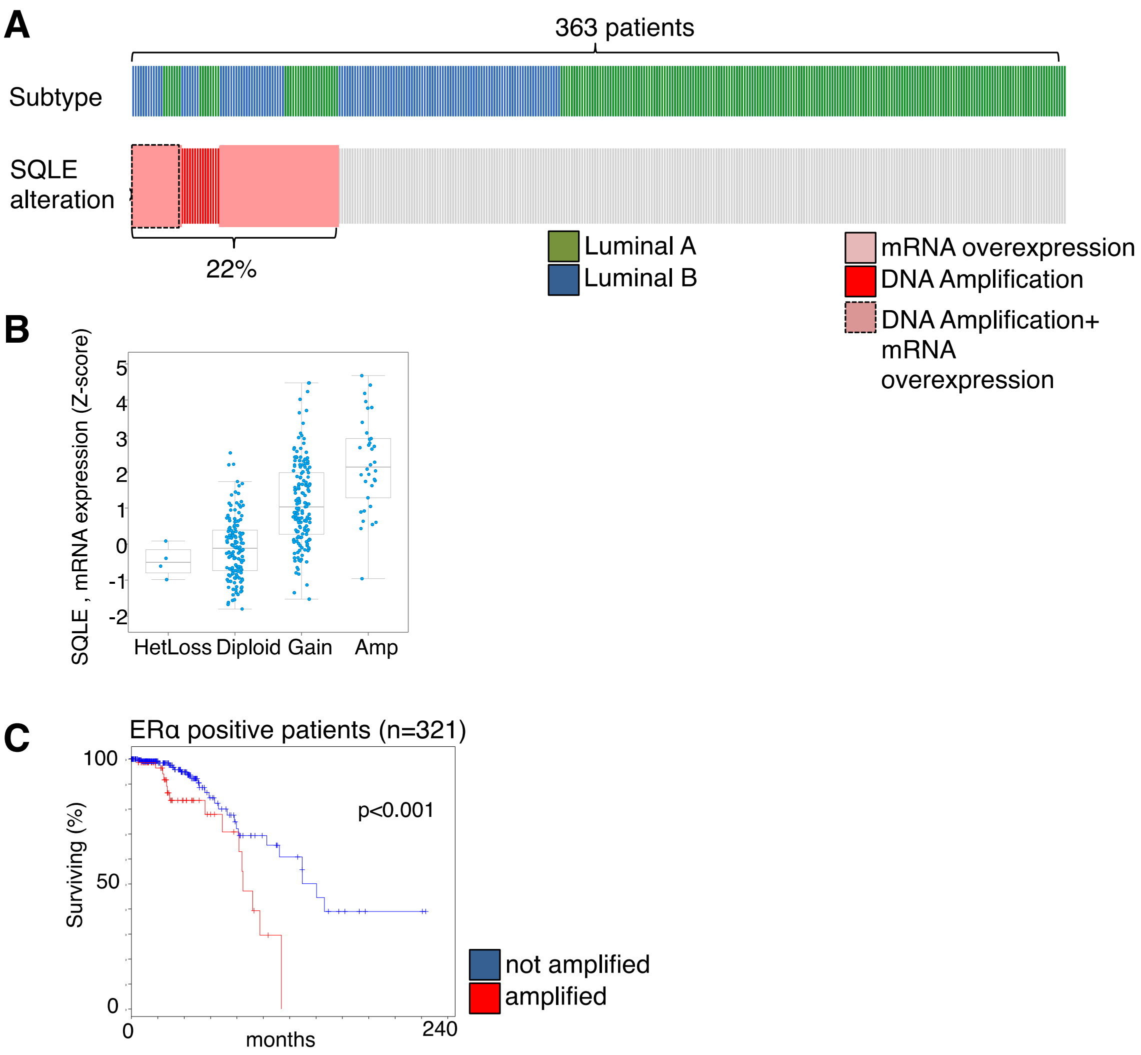

Supplementary Figure 10 A) *SQLE* copy number alteration within the TCGA cohort was calculated using cBioPortal. Stratification into luminal A or luminal B sub-groups is shown. B) Correlation plots for SQLE copy-number variations vs. RNA expression levels. C) Stratification of TCGA ERα patients using SQLE copy number/over-expression as a classifier.

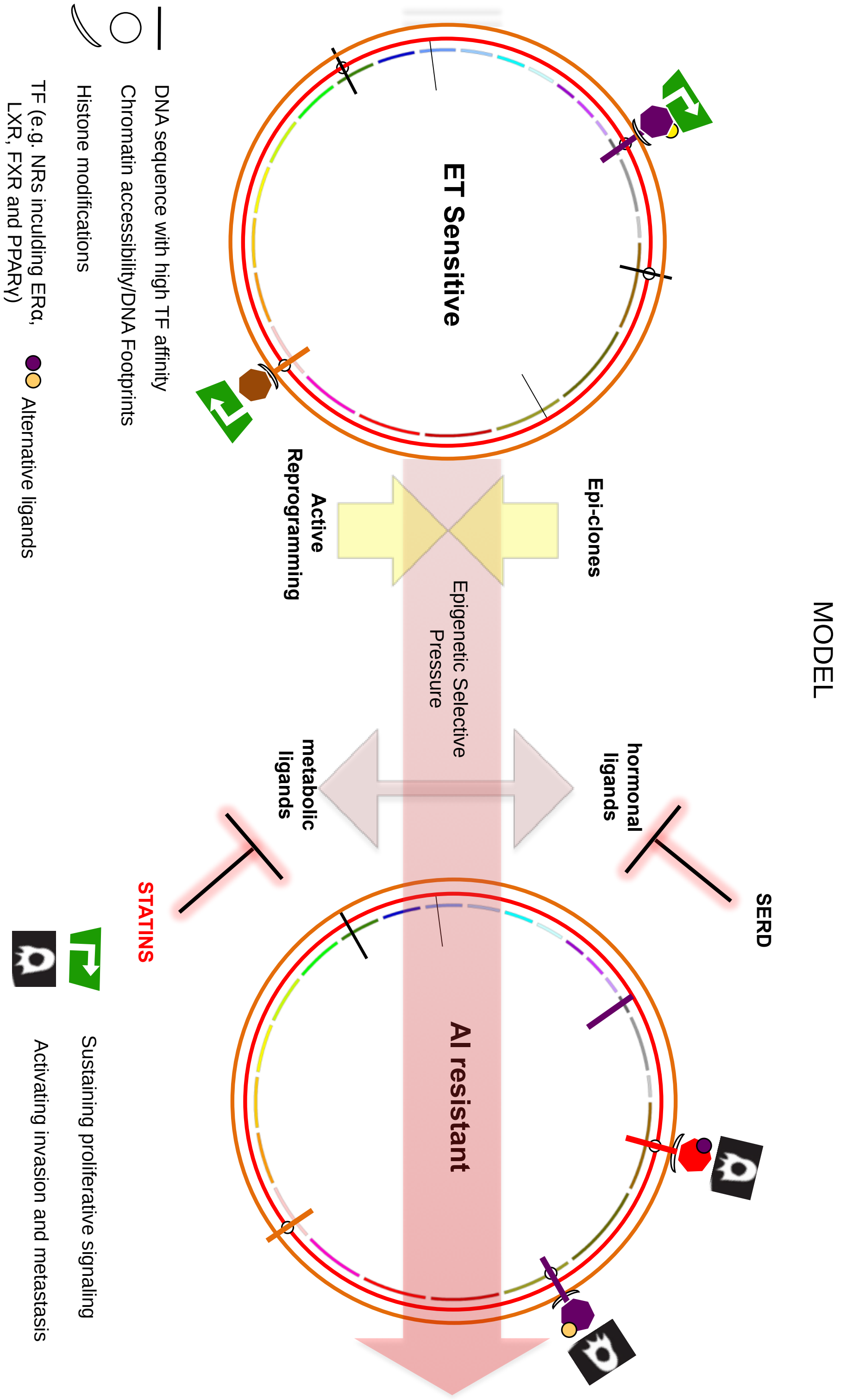

Supplementary Figure 11 Current working model. During the development of endocrine therapy resistance, cancer cells engage in extensive epigenetic reprogramming including histone modifications and chromatin accessibility. This leads to sustained

production of metabolic ligands and endogenous activation of ERα and possibly other nuclear receptors while promoting increased invasive potential.

Supplementary Methods

Construction of transcriptomics databases

Breast cancer datasets were identified in GEO (http://www.ncbi.nlm.nih.gov/gds) using the GEO platform IDs "GPL96" (for HG-U133A), "GPL570" (for HG-U133 Plus 2.0) and the keywords "breast", "cancer" and "survival". The database quality control and removal of duplicate samples was performed as described previously[3]. 660 patients have TP53 mutation annotation. 184 are have TP53 mutations and 62 of those are positive for estrogen receptor alpha.

Illumina gene chips of 1988 patients published in the METABRIC project were obtained from the European Genome-phenome Archive (EGA) (https://www.ebi.ac.uk/ega/)[4]. During processing, instead of using the normalized table, we re-ran the pre-processing for all gene chips. Raw data was imported into R and summarized using the beadarray package[5]. For annotation, the illuminaHumanv3 database was used. Quantile normalization was carried out using the preprocessCore package[6].

RNA-seq data for breast cancer patients[7] is published in The Cancer Genome Atlas (TCGA) of the National Cancer Institute (http://cancergenome.nih.gov/) and we downloaded the pre-processed level 3 data generated using the Illumina HiSeq 2000 RNA Sequencing Version 2 platform.

Survival analysis

Kaplan-Meier analysis was performed as described previously[8]. For the expression of the genes, the median expression was used as the cutoff in a Cox regression analysis. The Kaplan-Meier survival plot, and hazard ratio with 95% confidence intervals and logrank P value were calculated and plotted in R using Bioconductor packages.

ChIP-seq data analysis

Single-end 50nt long reads were obtained on a HiSeq 2000 instrument (Illumina) and aligned using Bowties with default settings [9]. IPs were compared to their respective cell-type specific input using MACS v1.4[10] with default settings (p-value<=1e-5). In order to visualize the raw profiles on the UCSC Genome Browser [10], wiggle files were generated with MACS v1.4 and converted to bigWig. Visualization via CHASE [11] was carried out with default settings. For the CHASE analysis, promoter regions were defined as those < 1kb from TSS (RefSeq annotation).

RNA sequencing and analysis

One microgram of total RNA from each sample was used to produce cDNA libraries with the TruSeq Stranded Total RNA Sample Prep Kit (Illumina) following the manufacturer's instructions. Paired-end sequences 100-nt long reads were generated using a HiSeq 2000 instrument (Illumina). Fastq files containing the sequence reads, obtained at the end of the sequencing, were mapped to the University of California at Santa Cruz (UCSC) human reference genome (hg19 assembly) as previously described[12]. The mapped bam files (obtained with TopHat version 1.4.1 http://ccb.jhu.edu/software/tophat/index.shtml) were cleaned from duplicates with Picard

tools version 1.81 (http://picard.sourceforge.net) and then analysed using Cufflinks version 2.0.2 (http://cufflinks.cbcb.umd.edu) for transcript assembly, quantification and differential expression analysis. For some follow-up analyses (association of genes to differentially H3K27ac regions or Super-Enhancers, see below) the FPKM information was collapsed from the level of RefSeq transcripts to that of single gene symbols. In the case of multiple entries, the one showing the highest FPKM was retained.

Correlation with ENCODE data

ENCODE (ENCODE Project Consortium, 2012) H3K27ac data for K562, HelaS3 and A549 cell lines (two replicates each) were downloaded from the UCSC genome browser[13] golden path (hg19 release of the human genome). Data include alignments (bam format) as well as calling of enriched regions (broadPeak format). Using custom scripts, these regions were pooled and, in the cases of overlap, their coordinates were merged with those of H3K27ac-enriched regions in at least one out of six breast cell lines.

For the ENCODE data as well as for the breast cancer lines, the coordinates of the aligned reads were extended to 200 bps and a maximum of PCR duplicates of 2 was allowed. CoverageBed[14] was then used to compute coverage, which was transformed to RPKM for each region and sample. Using R, the correlation matrix among samples was calculated (Spearman's Rank Correlation Coefficient) and hierarchically clustered using the hclust function.

Comparative ChIP-seq analysis

Comparisons were performed using MACS v1.4[10] with default parameters. The H3K27ac profile from each cell line was compared to MCF7 and vice-versa. A region was considered as differentially acetylated if showing a p-value <= $1e^{-10}$ in the comparison between IPs and, if overlapping, it was considered an acetylated region enriched versus the corresponding cell-type-specific input DNA (p-value <= $1e^{-5}$).

<u>H3K27ac co-variation analysis</u>

Differentially acetylated regions identified in the previous paragraph were pooled and their coordinates merged in case of overlap. CoverageBed[14] was then used to compute coverage, which was transformed to RPKM for each region and sample. RPKM were log2-transformed (after setting zeros to the lowest value except zero) and subjected to hierarchical clustering (average linkage) using 1 – Pearson's correlation as a measure of distance. This allowed us to group together active regulatory elements showing the same pattern of variation across the six conditions under evaluation. In order to identify discrete clusters from the resulting dendrogram, the dynamicTreeCut R package[15] was used (method set to hybrid and minClusterSize to 100).

The resulting regions were also annotated – using a custom script – as TSS-proximal or TSS-distal according to their proximity to the TSSs of RefSeq genes. All the regions whose central coordinates lay within 2.5 kbps of annotated TSSs were considered proximal. The remaining ones were considered distal. The proximal regions could be unambiguously assigned to the gene they possibly regulated and we could then retrieve the corresponding FPKMs from the RNA-seq data analysis.

For visualization purposes, the behaviours of clusters were shown as heatmaps, using the mean of the log2-RPKM of the H3K27ac in each cell line as representative. Considering the mRNA of the regulated genes, the median of the linear FPKM for each cluster and cell line was also shown as a heatmap.

Super-Enhancers calling, clustering and annotation

Super-Enhancer (SE) regions were called separately in each of the six cell lines considered, using a variation of a previously applied approach[16]. Regions within 2.5 kbps of RefSeq TSSs were excluded and the remaining regions were merged together if less than 12.5 kbps intervened in between. After that, coverageBed[14] was used to compute the H3K27ac reads coverage of each region. These were then sorted by decreasing the total number of reads, and the top 5% of regions were retained as SEs.

The lists of cell-type-specific SEs were then pooled and overlapping regions merged. This list was used in all the following analyses. First of all, they were clustered applying the same procedure as that described in the paragraph H3K27ac co-variation analysis, except for the minClusterSize that was set to 20. To annotate the regulatory activity of SE-clusters to RefSeq genes, two complementary approaches were used:

- all the transcriptional units overlapping the SE-cluster were defined as regulated by the cluster;
- the nearest transcriptional unit not overlapping the SE-cluster was also considered.

The retrieval of the FPKM values of these genes as well as the criteria used for visualization followed the same approaches as those described in the paragraph H3K27ac co-variation analysis.

Quantification of H3K27ac in Topologically Associated Domains

Topologically Associated Domains (TADs) from IMR90 cells[17] were downloaded from http://chromosome.sdsc.edu/mouse/hi-c/download.html as hg18 coordinates, which were lifted to hg19 using liftOver[13].

Each gene in the human genome was assigned its TAD, and the H3K27ac levels in each TAD were expressed as RPKM coverage. The Log2-ratio of H3K27ac among cell lines could then be computed for each TAD.

Motif enrichment analysis

SE-associated regions identified by H3K27ac range from a few kilobases to hundreds of kilobases. In order to focus the enrichment analyses for known motifs on DNA stretches more likely to represent clusters of TF-binding sites, we looked for depressions ("valleys") in the H3K27ac nucleosome patterns of each cell line. PeakSplitter[18] was run on the regions of interest providing the H3K27ac wiggle profile (options -c 5 -f -v 0.7). In this way, sub-peaks corresponding to nucleosome signals were identified. Valleys were defined as the average coordinate between the start and the end of two contiguous sub-peaks within 50 base pairs from each other. Motif enrichment analyses were run on lists of valleys that were extended 50 base pairs on each side.

The script findMotifsGenome.pl from the suite HOMER[19] was used to find enrichment for known motifs. For each item of the list of regions subjected to analysis (either a cluster of enhancer-promoters or of SE), we considered the valleys calculated on the profile of the cell line showing the highest mean H3K27ac level (in the cluster).

RefSeq genes version

The coordinates of the genes were downloaded from the UCSC genome browser[13] on 2012, May 10th.

Statistics and plots

All plots were drawn and statistics were performed using R. Heatmaps were drawn using the heatmap.2 function of the gplots package. Statistics for expression data from FEMARA trial were conducted using Prism (non-paramentric Mann-Whitney U test).

Supplementary References

1. Magnani, L., Ballantyne, E. B., Zhang, X. & Lupien, M. PBX1 genomic pioneer function drives ERα signaling underlying progression in breast cancer. *PLoS Genet* **7,** e1002368 (2011).
2. Rhodes, D. R. *et al.* Mining for regulatory programs in the cancer transcriptome. *Nat Genet* **37,** 579–583 (2005).
3. Györffy, B. & Schäfer, R. Meta-analysis of gene expression profiles related to relapse-free survival in 1,079 breast cancer patients. *Breast Cancer Res Treat* **118,** 433–441 (2009).
4. Curtis, C. *et al.* The genomic and transcriptomic architecture of 2,000 breast tumours reveals novel subgroups. *Nature* **486,** 346–352 (2012).
5. Dunning, M. J., Smith, M. L., Ritchie, M. E. & Tavaré, S. beadarray: R classes and methods for Illumina bead-based data. *Bioinformatics (Oxford, England)* **23,** 2183–2184 (2007).
6. Bolstad, B. M., Irizarry, R. A., Astrand, M. & Speed, T. P. A comparison of normalization methods for high density oligonucleotide array data based on variance and bias. *Bioinformatics (Oxford, England)* **19,** 185–193 (2003).
7. Cancer Genome Atlas Network. Comprehensive molecular portraits of human breast tumours. *Nature* **490,** 61–70 (2012).
8. Györffy, B., Lanczky, A. & Szallasi, Z. Implementing an online tool for genome-wide validation of survival-associated biomarkers in ovarian-cancer using microarray data from 1287 patients. *Endocr Relat Cancer* **19,** 197–208 (2012).
9. Langmead, B., Trapnell, C., Pop, M. & Salzberg, S. L. Ultrafast and memory-efficient alignment of short DNA sequences to the human genome. *Genome Biol* **10,** R25 (2009).
10. Zhang, Y. *et al.* Model-based analysis of ChIP-Seq (MACS). *Genome Biol* **9,** R137 (2008).
11. Nielsen, C. B. *et al.* Spark: a navigational paradigm for genomic data exploration. *Genome Res* **22,** 2262–2269 (2012).
12. Pinho, F. G. *et al.* Downregulation of microRNA-515-5p by the estrogen receptor modulates sphingosine kinase 1 and breast cancer cell proliferation. *Cancer Res* **73,** 5936–5948 (2013).
13. Fujita, P. A. *et al.* The UCSC Genome Browser database: update 2011. *Nucleic Acids Res* **39,** D876–82 (2011).
14. Quinlan, A. R. & Hall, I. M. BEDTools: a flexible suite of utilities for comparing genomic features. *Bioinformatics* **26,** 841–842 (2010).
15. Langfelder, P., Zhang, B. & Horvath, S. Defining clusters from a hierarchical cluster tree: the Dynamic Tree Cut package for R. *Bioinformatics (Oxford, England)* **24,** 719–720 (2008).
16. Hnisz, D. *et al.* Super-Enhancers in the Control of Cell Identity and Disease. *Cell* (2013). doi:10.1016/j.cell.2013.09.053
17. Dixon, J. R. *et al.* Topological domains in mammalian genomes identified by analysis of chromatin interactions. *Nature* **485,** 376–380 (2012).
18. Salmon-Divon, M., Dvinge, H., Tammoja, K. & Bertone, P. PeakAnalyzer: genome-wide annotation of chromatin binding and modification loci. *BMC*

Bioinformatics **11,** 415 (2010).

19. Heinz, S. *et al*. Simple combinations of lineage-determining transcription factors prime cis-regulatory elements required for macrophage and B cell identities. *Mol Cell* **38,** 576–589 (2010).
